# Mitochondrial and Cellular Function in Fibroblasts, Induced Neurons, and Astrocytes Derived from Case Study Patients: Insights into Major Depression as a Mitochondria-Associated Disease

**DOI:** 10.3390/ijms25020963

**Published:** 2024-01-12

**Authors:** Iseline Cardon, Sonja Grobecker, Selin Kücükoktay, Stefanie Bader, Tatjana Jahner, Caroline Nothdurfter, Kevin Koschitzki, Mark Berneburg, Bernhard H. F. Weber, Heidi Stöhr, Marcus Höring, Gerhard Liebisch, Frank Braun, Tanja Rothammer-Hampl, Markus J. Riemenschneider, Rainer Rupprecht, Vladimir M. Milenkovic, Christian H. Wetzel

**Affiliations:** 1 Department of Psychiatry and Psychotherapy, University of Regensburg, 93053 Regensburg, Germany; iseline.cardon@ukr.de (I.C.);; 2 Department of Dermatology, Regensburg University Hospital, 93053 Regensburg, Germany; 3 Institute of Human Genetics, University of Regensburg, 93053 Regensburg, Germany; 4 Institute of Clinical Human Genetics, Regensburg University Hospital, 93053 Regensburg, Germany; 5 Institute of Clinical Chemistry and Laboratory Medicine, Regensburg University Hospital, 93053 Regensburg, Germany; 6 Department of Neuropathology, Regensburg University Hospital, 93053 Regensburg, Germany

**Keywords:** major depressive disorder, mitochondrial functions, mitochondriopathy, treatment-resistant depression, iPS-neurons, iPS-astrocytes

## Abstract

The link between mitochondria and major depressive disorder (MDD) is increasingly evident, underscored both by mitochondria’s involvement in many mechanisms identified in depression and the high prevalence of MDD in individuals with mitochondrial disorders. Mitochondrial functions and energy metabolism are increasingly considered to be involved in MDD’s pathogenesis. This study focused on cellular and mitochondrial (dys)function in two atypical cases: an antidepressant non-responding MDD patient (“Non-R”) and another with an unexplained mitochondrial disorder (“Mito”). Skin biopsies from these patients and controls were used to generate various cell types, including astrocytes and neurons, and cellular and mitochondrial functions were analyzed. Similarities were observed between the Mito patient and a broader MDD cohort, including decreased respiration and mitochondrial function. Conversely, the Non-R patient exhibited increased respiratory rates, mitochondrial calcium, and resting membrane potential. In conclusion, the Non-R patient’s data offered a new perspective on MDD, suggesting a detrimental imbalance in mitochondrial and cellular processes, rather than simply reduced functions. Meanwhile, the Mito patient’s data revealed the extensive effects of mitochondrial dysfunctions on cellular functions, potentially highlighting new MDD-associated impairments. Together, these case studies enhance our comprehension of MDD.

## 1. Introduction

### 1.1. Epidemiology of MDD

At present, an estimated 3.8% of the global population (280 million people) are living with a depressive disorder [1]. Among them, 193 million people suffered from major depressive disorder (MDD), and this number has surged by 26% as a result of the COVID-19 pandemic, reaching a staggering 246 million people. Therefore, studying MDD appears increasingly relevant. MDD is a complex condition with multiple factors and a genetic basis, emerging from an intricate interplay of vulnerability genes and environmental factors that accumulate influence over an individual’s lifetime. Stressful life experiences, particularly those encountered early in life, are proposed to play a pivotal role in influencing brain development. This influence can result in enduring functional changes, potentially contributing to a lifelong susceptibility to mental health afflictions (for review, see [2]). 

### 1.2. Pathophysiological Hypotheses

Although the precise pathomechanisms underlying MDD development are still not completely understood, various hypotheses have been put forward. One of the oldest and widely accepted hypotheses describes the dysregulation of neurotransmission, especially in the monoaminergic system. Given that treatments restoring monoamines levels within hours only alleviate symptoms after several weeks, some argue that MDD is mainly a result of reduced neuroplasticity resulting from impaired BDNF signaling [3,4]. In line with this theory, Casarotto et al. showed that common antidepressants (ADs) likely exert their clinical effects through their binding to the neurotrophins receptor TRKB [3]. Yet, for primary AD treatments, the remission rate ranges between 30 and 45% [5]. The mechanisms of AD resistance are not completely clear, but a number of predictors for AD response have been identified [6].

Numerous studies link depression and inflammation. MDD patients show elevated peripheral inflammatory factors [7,8]. Inflammation can be caused by stress [8], and stress can result in disturbances in the hypothalamus–pituitary–adrenal axis (HPA), which is strongly associated with MDD [9]. HPA hormones like cortisol can, in turn, affect plasticity, potentially contributing to the development of depression [10]. Understanding these pathways and how they interconnect and participate with the pathophysiology of MDD is crucial for developing new treatments. 

### 1.3. Mitochondrial Dysfunction in MDD

Mitochondria and energy metabolism have turned into focus in the pathomechanisms of depression [11,12,13,14]. Mitochondrial dysfunction, resulting in decreased energetic capacity, increased oxidative stress, and alterations in signaling, is regarded as a key risk factor for MDD and other psychiatric disorders [12,13,14,15]. Neurons rely heavily on a consistent supply of energy for their proper physiological function. Remarkably, it is estimated that 75% of the total adenosine triphosphate (ATP) consumption in the brain serves to maintain resting membrane potential, which is critical for membrane excitability and neurotransmission [16]. Consequently, neurons are very susceptible to metabolic stress, which can be caused by mitochondrial dysfunction. 

### 1.4. Mitochondrial Diseases

Mitochondrial diseases (MDs) result from dysfunctional mitochondria and form a group of clinically heterogeneous genetic disorders. MDs are far more frequent than previously assumed. Schaefer et al. estimated a prevalence of 9.2 mitochondrial DNA diseases per 100,000 adults [17]. In children below 16 years of age, the estimated prevalence of MDs ranges from 5 to 15 cases per 100,000 individuals [18]. However, mitochondrial disorders are hard to define in children and induce unspecific symptoms, making misdiagnoses likely and possibly resulting in underdiagnosed MDs [19]. Predominantly, MDs lead to defects in oxidative phosphorylation. Such shortcomings can affect any tissue, although those requiring high levels of energy, such as muscle and brain, are most severely affected. Symptoms can encompass non-neurological or neurological manifestations and typically involve multiple organ systems [18].

The most commonly affected structure in MDs is the nervous system, with symptoms including stroke-like episodes, migraine, epilepsy, spasticity and ataxia, visual impairment, hearing loss, intellectual disability, and fluctuating encephalopathy [20]. Brain dysfunction in MDs can also lead to neuropsychological or psychiatric disturbances. Indeed, Morava et al. showed that 70% of MD patients will experience a major mental illness at some point during their lives [21]. Moreover, Fattal et al. reported depressive behavior in 50% of children with an MD [22]. 

### 1.5. Mitochondrial Diseases and Psychiatric Illnesses

Interestingly, in a case series on MD, Anglin et al. reported that 11 out of 12 patients presented treatment-resistant psychiatric illnesses [23]. Furthermore, clinical deterioration upon treatment with psychotropic medication has been shown in patients with MDs [23,24]. Many psychotropic drugs are known to impair mitochondrial functions [25]; although, as Riquin et al. pointed out, “it is challenging to delineate whether mitochondrial dysfunction occurs secondary to pharmaceutical treatment or whether it is a result of the underlying disease process itself” [24]. These observations highlight the need to consider MDs in patients diagnosed with psychiatric illnesses, such as MDD, and to adapt treatment accordingly.

### 1.6. Novel Cellular Model Approach

On the one hand, neurons are highly vulnerable to metabolic stress, which can lead to psychiatric illness such as MDD. On the other hand, mitochondrial impairments associated with MDD have been reported in peripheral cells such as muscle cells [26], platelets [27,28], peripheral blood mononuclear cells [29], and fibroblasts [30,31]. As a result, it is becoming increasingly clear that MDD is not merely limited to mental afflictions but also encompasses physical manifestations. This emphasizes the importance to consider MDD-associated pathomechanisms in neuronal as well as non-neuronal cells. Therefore, we studied a human cellular model of MDD in order to unravel the molecular pathomechanisms related to mitochondrial dysfunction and bioenergetics imbalance. A model consisting of various types of patient-derived cells represents a unique opportunity to recapitulate many cellular pathophysiological features of MDD as a human-specific disorder.

In a previous study, we collected dermal fibroblasts from MDD patients and non-depressed controls and demonstrated a clear mitochondrial impairment, including reduced respiration and ATP content [31]. We then reprogrammed the fibroblasts to induced pluripotent stem cells (iPSCs), which we subsequently differentiated from neural progenitor cells (NPCs) and neurons [32]. Like in peripheral cells, we observed signs of altered mitochondrial function in the neural lineage including lower respiration rates. The reduced OXPHOS and altered bioenergetic properties most likely contributed to altered physiological function and support our findings in neurons. Indeed, electrophysiological measurements showed significantly lower membrane capacitance, a more depolarized membrane potential, and increased spontaneous electrical activity [32]. Moreover, astrocyte pathology has been reported in human post-mortem brain tissue of MDD patients [33], suggesting that these glial cells also play a role in the etiology of depression. Therefore, in the present study, we extended our human depression model to iPSC-derived astrocytes.

### 1.7. Complementing Cohort Studies with Case Studies

The aim of the present study was to build on the above-mentioned cohort studies and complement them with case studies in order to extend our knowledge on the biological mechanisms underpinning the development of MDD [31,32]. Case studies are detailed, intensive studies that seek to explain particular cases and understand causal mechanisms and processes. They allow an in-depth understanding of the factors under study. The focus on outlier cases that do not fit the general theory brings the potential to help understand mechanisms that had not been explored before. Where cohort studies measure a lot of cases with a limited number of parameters, case studies aim at measuring a few cases very precisely to understand the interplay between different parameters. 

### 1.8. Presentation of the Case Study Patients

Here, we investigated an antidepressant (AD) non-responder MDD patient and a non-depressed mitochondriopathy patient in order to gain new perspectives on MDD in the context of the bioenergetics hypothesis of psychiatric disorders.

The first patient was defined as AD non-responder after three treatment regimens failed to induce remission over twelve weeks, according to the most accepted definition of treatment-resistant depression (TRD) (see Table 1 for patient information) [6,34]. In contrast with patients from the MDD cohort who all achieved a certain degree of remission (average remission 58 ± 7%), the Non-R patient still suffered from severe MDD at the time of the biopsy. Studying this patient was therefore an opportunity to investigate how cellular and mitochondrial functions differed between remitted, treatment-responding MDD patients and a severely depressed, treatment-resistant MDD patient.

The mitochondriopathy patient presented several neurological symptoms, somatic symptoms, and physical pains. She was directed to the Center for Rare Diseases, Regensburg, Germany, where a series of tests allowed to rule out several pathologies and disorders (see Table 1 for patient information). As certain symptoms pointed toward a mitochondrial disorder, exome and mitochondrial genome were sequenced and showed no mitochondrial disease-associated mutations. However, blood tests revealed strong alterations in several factors that play a critical role in mitochondrial function. Studying this patient was an opportunity to observe the interplay between different cellular and mitochondrial parameters and compare them in the context of mitochondriopathy and of MDD. A further aim was to understand how specific cellular processes and characteristics that could be altered in depression were influenced by mitochondrial (dys)function. This case study was therefore relevant to our understanding of different aspects of MDD pathophysiology related to mitochondria.

We investigated cellular and mitochondrial functions in fibroblasts, NPCs, astrocytes, and neurons derived from these two patients and their matched healthy controls. In those different cell types, bioenergetic functions were assessed by measuring mitochondrial respiration, ATP content, substrate availability, mitochondrial content, cell size, and mitochondrial membrane potential. Mitochondrial and cytosolic Ca^2+^ levels were measured. Oxidative stress was investigated by measuring mitochondrial and cellular reactive oxygen species (ROS), lipid peroxidation, and antioxidant capacity of the glutathione system. Moreover, we analyzed electrophysiological properties of neurons using whole-cell voltage- and current-clamp measurements.

The results presented here help to refine our understanding of the role of mitochondrial dysfunction in MDD and may contribute to the development of new, or modification of existing, therapies.

## 2. Results

To investigate the neurobiological underpinnings of major depressive disorder (MDD), we are studying cellular and mitochondrial function in human cellular models. We characterized dermal fibroblasts [31] and generated induced pluripotent stem cells (iPSCs), which we further differentiated to neural progenitor cells (NPCs) and induced neurons [32]. Human cellular models are particularly valuable for studying a complex multifactorial disorder like MDD while preserving the genetic and epigenetic signatures of individuals. We examined peripheral and central cell types to capture the systemic nature of MDD, which encompasses somatic manifestations outside the brain. To extend our knowledge on the involvement of mitochondrial dysfunction in MDD, the present work focused on two specific patients, one suffering from a suspected mitochondriopathy (“Mito”) and one antidepressant non-responding MDD patient (“Non-R”). The results from these case study patients are directly compared to respective controls and to changes observed between MDD and control cohorts [31,32].

Further information on MDD study participants can be found in the Methods and in [31,32]. Exome sequencing of the Mito and Non-R patient fibroblast gDNA did not reveal any known disease-associated variants (point mutations or indels). 

### 2.1. Fibroblasts from the Antidepressant Non-Responder (Non-R) and the Mitochondriopathy Patient (Mito) Show Altered Bioenergetic Properties and Calcium Homeostasis

#### 2.1.1. Bioenergetic Properties

**Figure 1:**
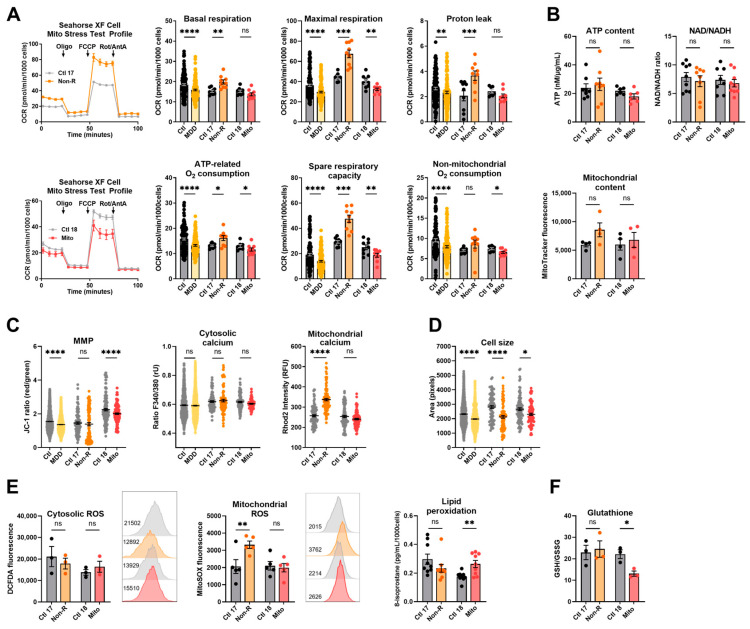
Mitochondrial bioenergetics in fibroblasts. (**A**) Mitochondrial respiration. The oxygen consumption rate (OCR) was measured following the Agilent XF Mito Stress Test protocol consisting of sequential injections of oligomycin (Oligo), carbonyl cyanide-4-(trifluoromethoxy)-phenylhydrazone (FCCP), and rotenone/antimycin A (Rot/AntA) to reveal different respiratory parameters. **Left**: representative OCR curves for Ctl 17/Non-R (above) and Ctl 18/Mito (below). **Right**: OCR of control and patient’s fibroblasts in key respiratory parameters. Bar plots show normalized mean OCR values ± SEM. (**B**) Bioenergetic parameters. ATP content was measured using a luminescent assay and normalized to protein amount. Bar plot shows nM ATP per µg/mL proteins ± SEM. Substrate availability was estimated by measuring the NAD/NADH ratio with a colorimetric assay. Bar plots represent mean NAD/NADH ratio ± SEM. Mitochondrial content was measured using flow cytometry and is indicated by MitoTracker Green mean fluorescence ± SEM, and 2 × 10^4^ events were recorded for each replicate. (**C**) MMP and calcium homeostasis. MMP was measured with the JC-1 dye and is indicated by the fluorescence ratio between JC-1 aggregates and JC-1 monomers. Dot plot shows mean red/green ratios ± SEM. Cytosolic calcium was measured as the Fura-2 fluorescence ratio F340/380 and is represented as mean ratio ± SEM. Mitochondrial calcium levels were measured using Rhod-2/AM and is presented as mean fluorescence intensity, in relative fluorescent unit ± SEM. (**D**) Cell size was analyzed by assessing area (pixels) of Fura-2/AM-loaded cells. Dot plot shows the number of pixels ± SEM. (**E**) Oxidative stress indicators. Cytosolic reactive oxygen species (ROS) and mitochondrial ROS (superoxide) were measured using flow cytometry and are indicated by DCFDA and MitoSOX mean fluorescence, respectively. Bar plots show mean fluorescence ± SEM, and 2 × 10^4^ events were recorded for each replicate. On the right of each graph, with matching color coding and order, are representative histograms showing fluorescence (x-axis) and cell count (y-axis). Numbers indicate mean fluorescence. Lipid peroxidation was estimated by measuring 8-isoprostane concentration in cell culture supernatants and normalized to the number of cells. Bar plot shows mean 8-isoprostane concentration in pg/mL/1000 cells ± SEM. (**F**) Antioxidant system function was estimated with the ratio of reduced (GSH) to oxidized (GSSG) glutathione using a luminescent assay. Bar plots show the mean ratio GSH/GSSG ± SEM. Ctl: non-depressed controls cohort (grey); MDD: major depressive disorder cohort (yellow); Ctl 17 and Ctl 18: non-depressed controls (grey); Non-R: non-responder patient (orange); and Mito: mitochondriopathy patient (red). All data were analyzed with paired *t*-test, and significant differences were indicated with * (*p* < 0.05), ** (*p* < 0.005), *** (*p* < 0.0005), and **** (*p* < 0.0001). Mean, SEM, *n*, and *p*-value are compiled in Table S1.

To investigate the activity of the oxidative phosphorylation system (OXPHOS) as a key function of mitochondrial metabolism, we measured the oxygen consumption rate (OCR) in the fibroblasts of patients and of sex- and age-matched controls. In the mitochondriopathy patient (Mito), OCR was significantly decreased across most assessed parameters. Likewise, patients from the MDD cohort exhibited a lower OCR in most parameters (Figure 1A, Table S1), which aligns with alterations reported in MDD patients’ cells in the literature. This similarity suggests that reduced mitochondrial respiration is a core characteristic in both mitochondriopathy and MDD and might be related to the symptoms of fatigue observed in these disorders. Surprisingly, the antidepressant non-responder patient (Non-R) showed markedly increased maximal respiration, spare respiratory capacity, and proton leak when compared to its matched control and to the MDD and control cohort (Figure 1A, Table S1). These results challenge the theory that mitochondrial dysfunction in MDD is solely about reduced functions and rather indicate excessively increased respiration rates.

Although OXPHOS activity significantly varies in patient fibroblasts, we did not detect differences in the cellular content of ATP, the NAD/NADH ratio, and the mitochondrial content (Figure 1B, Table S1).

The mitochondrial membrane potential (MMP) is indicative of the proton motive force and indirectly reflects the metabolic activity and capacity of mitochondria. It can be assessed by labeling mitochondria with the potential-dependent ratiometric dye JC-1. Like the MDD patients, the Mito patient showed a lower MMP than the non-depressed control (Figure 1C, Table S1). However, despite highly increased respiration, the MMP was not significantly different in the Non-R patient (Figure 1C, Table S1). Together with unchanged ATP, this suggests a loss of proton motive force and indicates that heightened respiration does not necessarily reflect better mitochondrial function in this MDD patient.

#### 2.1.2. Calcium Homeostasis and Cell Size

The cellular Ca^2+^ homeostasis is affected by energy-demanding active transport processes and dependent on chemical and electrical gradients. Ca^2+^ levels also have regulatory effects on signaling, enzyme function, and metabolism. Using the Ca^2+^-sensitive dyes Fura-2/AM and Rhod-2/AM, we investigated cytosolic and mitochondrial Ca^2+^ levels, respectively. Consistent with results from the MDD cohort fibroblasts, no significant differences were observed in the cytosolic Ca^2+^ levels in our case study patients (Figure 1C, Table S1). However, alongside increased respiration, a marked increase in mitochondrial Ca^2+^ was found in the Non-R patient, while there was no change in the Mito patient (Figure 1C, Table S1). Considering the activating role of Ca^2+^ on OXPHOS, this increase could participate in the high respiratory rates observed in Non-R patient’s fibroblasts.

Like in the MDD cohort, the patients’ fibroblasts were significantly smaller than the control cells when assessed in Fura-2/AM-loaded cells (Figure 1D, Table S1). This finding is consistent with the hypothesis of a metabolic/bioenergetic dysregulation in patient cells, which could also lead to morphological changes.

A putative relationship between the clinical severity of depressive symptoms and the biological findings was investigated in fibroblasts from the MDD cohort using linear regression models (Table S2). A negative correlation was observed between the Hamilton Depression Rating score and two respiratory parameters: maximal respiration and spare respiratory capacity (Figure S1). This suggests a parallel between the severity of mitochondrial respiration impairments and the clinical severity of depression in patients.

### 2.2. Redox Homeostasis Is Partly Affected in AD Non-R and Mito Patients’ Fibroblasts

Reactive oxygen species (ROS) exert important signaling functions but also cause oxidative stress leading to molecular damage. Mitochondria are major players in the maintenance of the cell’s redox homeostasis by generating ROS at complexes I and III of the electron transport chain (ETC). ROS also occur in the cytosol, where they can cause lipid peroxidation. The redox balance is maintained by the antioxidant system, including glutathione. 

We measured cytosolic ROS and mitochondrial superoxide via flow cytometry using the DCFDA and MitoSOX dyes, respectively. No significant difference appeared in the cytosolic ROS content of fibroblasts, but mitochondrial superoxide levels were significantly increased in fibroblasts from the Non-R patient (Figure 1E, Table S1). Such an increase is consistent with the hyperactive ETC observed in the respirometry experiments (Figure 1A, Table S1).

Lipid peroxidation was estimated by measuring one of its main by-products, 8-isoprostane, and was shown to be significantly increased in fibroblasts from the Mito patient. Assessing the glutathione oxidation with a luminescence-based kit revealed a significantly lower GSH/GSSG ratio in the Mito patient’s fibroblasts. This suggests a potential oxidative stress and lower antioxidant capacity in the Mito patient’s cells (Figure 1F, Table S1). Notably, oxidative stress and lipid peroxidation have been reported in skin fibroblasts from patients with mitochondrial disorders [35,36].

A broader analysis of cellular and mitochondrial lipids using an untargeted lipidomics approach did not reveal any marked alterations in the lipid composition of Mito or Non-R patient cells (identification and quantification of detected lipid species will be provided on request).

Taken together, findings in our case study patients’ cells indicated that Non-R fibroblasts have a strongly increased mitochondrial respiration along with increased mitochondrial Ca^2+^ levels and increased mitochondrial superoxide. Unchanged mitochondrial content indicated that these findings did not result from increased mitochondrial mass. The Mito patient’s fibroblasts had decreased respiration and decreased MMP, suggesting a bioenergetics impairment. Moreover, increased lipid peroxidation along with lower glutathione suggested a disturbed redox homeostasis. Cell size was decreased in both patients. 

### 2.3. Induced Neural Progenitor Cells of Patients Show Alterations in Bioenergetic Properties

**Figure 2:**
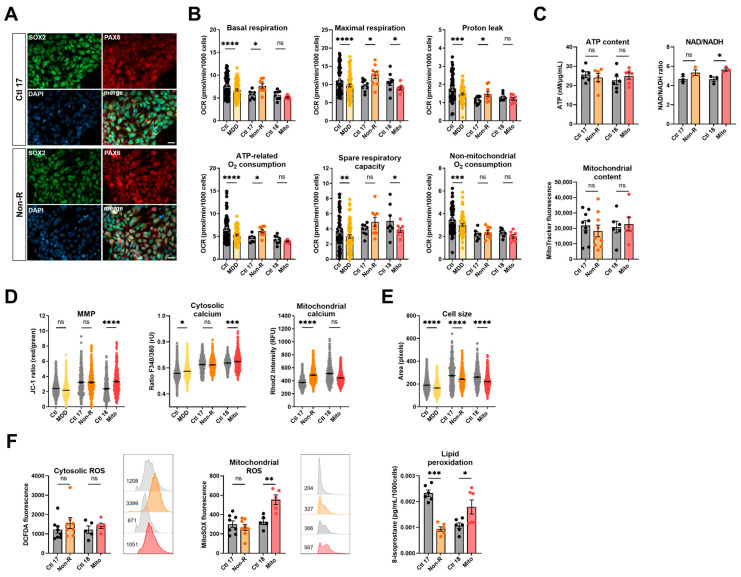
Mitochondrial bioenergetics in neural progenitor cells (NPCs). (**A**) Typical NPC markers. Representative images show PAX6 and SOX2 are co-expressed by a majority of the NPCs differentiated from Ctl 17 and Non-R patient (see Table S3 for quantification). Scale bar indicates 20 µm. (**B**) Mitochondrial respiration. The oxygen consumption rate (OCR) was measured following the Agilent XF Mito Stress Test protocol. OCR of control and patient NPCs in key respiratory parameters are represented with bar plots of normalized mean OCR values ± SEM. (**C**) Bioenergetic parameters. ATP content was measured using a luminescent assay and normalized to protein amount, and bar plot shows nM ATP per µg/mL proteins ± SEM. Substrate availability was estimated by measuring the NAD/NADH ratio with a colorimetric assay. Bar plots represent mean NAD/NADH ratio ± SEM. Mitochondrial content was measured using flow cytometry and is indicated by MitoTracker Green mean fluorescence ± SEM, and 1 × 10^5^ events were recorded for each replicate. (**D**) MMP and calcium homeostasis. *MMP* was measured with the JC-1 dye and is indicated by the fluorescence ratio between JC-1 aggregates and JC-1 monomers. Dot plot shows mean red/green ratios ± SEM. Cytosolic calcium was measured as the Fura-2 fluorescence ratio F340/380 and is represented as mean ratio ± SEM. Mitochondrial calcium levels were measured using Rhod-2/AM and is presented as mean fluorescence intensity, in relative fluorescent unit ± SEM. (**E**) Cell size was analyzed by assessing area (pixels) of Fura-2/AM-loaded cells. Dot plot shows the number of pixels ± SEM. (**F**) Oxidative stress indicators. Cytosolic reactive oxygen species (ROS) and mitochondrial ROS (superoxide) were measured using flow cytometry and are indicated by DCFDA and MitoSOX mean fluorescence, respectively. Bar plots show mean fluorescence ± SEM, and 1 × 10^5^ events were recorded for each replicate. On the right of each graph, with matching color coding and order, are representative histograms showing fluorescence (x-axis) and cell count (y-axis). Numbers indicate mean fluorescence. Lipid peroxidation was estimated by measuring 8-isoprostane concentration in cell culture supernatants and normalized to the number of cells. Bar plots show mean 8-isoprostane concentration in pg/mL/1000 cells ± SEM. Ctl: non-depressed controls cohort (grey); MDD: major depressive disorder cohort (yellow); Ctl 17 and Ctl 18: non-depressed controls (grey); Non-R: non-responder patient (orange); and Mito: mitochondriopathy patient (red). All data were analyzed with paired *t*-test, and significant differences were indicated with * (*p* < 0.05), ** (*p* < 0.005), *** (*p* < 0.0005), and **** (*p* < 0.0001). Mean, SEM, *n*, and *p*-value are compiled in Table S4.

To further investigate bioenergetic properties of neural cells, we used the patients’ primary skin fibroblasts and reprogrammed them to iPSCs via transient episomal transduction according to the Yamanaka protocol [37,38]. iPSCs were differentiated to NPCs and stained for the neural progenitor markers SOX2 and PAX6 [39] (Figure 2A). Table S3 shows that most cells co-express both markers.

#### 2.3.1. Bioenergetic Properties

NPCs from the MDD cohort had significantly lower respiration in all parameters measured (Figure 2B, Table S4). Similarly, NPCs from the Mito patient had lower OCR, with a significant decrease in maximal respiration and spare respiratory capacity (Figure 2B, Table S4). Like in fibroblasts, NPCs from the Non-R patient showed significantly increased OCR in basal and maximal respiration, increased proton leak, and ATP-related oxygen consumption. 

Cellular ATP levels and mitochondrial content were not significantly different in the NPCs of patients and their respective controls (Figure 2C, Table S4). However, an NAD/NADH ratio elevation was identified in both patients, yet statistical significance was reached only in the Mito patient (Figure 2C, Table S4), indicating that substrate availability might be affected in patients’ NPCs. In NPCs of the Non-R patient and the MDD cohort, the MMP did not significantly differ from the controls. However, a significant increase was observed between the Mito patient and its control. 

#### 2.3.2. Calcium Homeostasis and Cell Size

Cytoplasmic Ca^2+^ levels in NPCs were higher in both the Mito patient and the MDD cohort, while they did not differ between the Non-R patient and Ctl 17. In contrast, mitochondrial Ca^2+^ levels were higher in the Non-R patient NPCs and lower in the Mito patient (Figure 2D, Table S4). This is consistent with changes observed in mitochondrial respiration and suggests an interaction between Ca^2+^ homeostasis and bioenergetics in the patients’ cells. 

Consistent with observations in fibroblasts, NPCs were significantly smaller in the Mito and Non-R patients and in the MDD cohort, when compared to their controls (Figure 2E, Table S4).

#### 2.3.3. Redox Homeostasis

While there was no clear difference in cellular ROS between NPCs of patients and controls, as observed in fibroblasts, NPCs from the Mito patient showed a significant increase in mitochondrial superoxide (Figure 2F, Table S4). Despite a high interindividual variability between the controls, potentially attributable to the age difference (see Table 1 for patients’ information) [40,41], it appears that lipid peroxidation was decreased in the NPCs of the Non-R patient and increased in the Mito patient (Figure 2F, Table S4).

Taken together, findings in patients’ NPCs mostly aligned with results in fibroblasts. Non-R patient’s NPCs had higher respiration rates and mitochondrial Ca^2+^ levels. Mito patient’s NPCs had lower respiration but increased MMP and cytosolic Ca^2+^. Oxidative stress seemed increased. In both patients, NPCs were smaller. 

### 2.4. Induced Astrocytes of Patients Show Altered Bioenergetic Properties and Oxidative Stress

**Figure 3:**
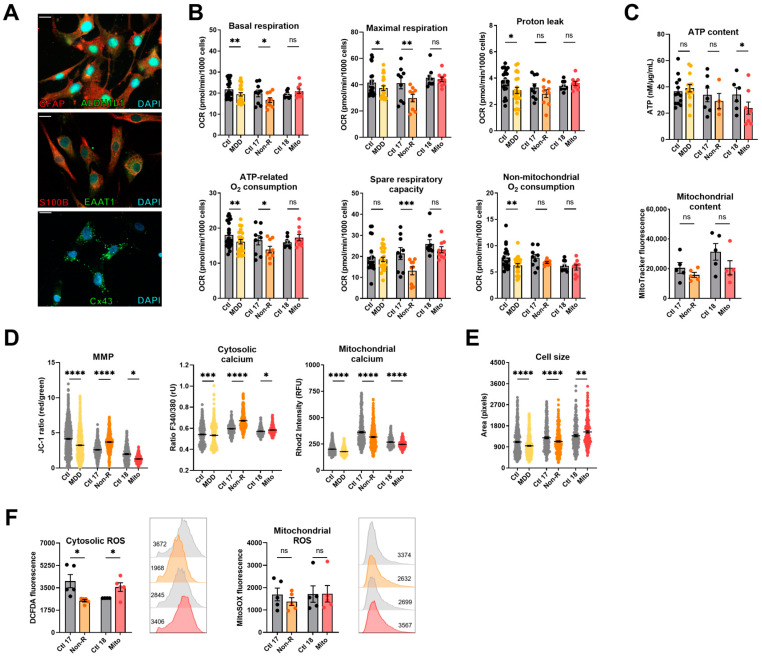
Mitochondrial bioenergetics in astrocytes. (**A**) Astrocyte markers. Immunofluorescence stainings show that cells express the typical mature astrocytes markers GFAP, ALDH1L1, S100β, EAAT1, and connexin 43. Scale bar indicates 20 µm. (**B**) Mitochondrial respiration. The oxygen consumption rate (OCR) was measured following the Agilent XF Mito Stress Test protocol. OCR of control and patient astrocytes in key respiratory parameters. Bar plots show normalized mean OCR values ± SEM. (**C**) Bioenergetic parameters. ATP content was measured using a luminescent assay and normalized to protein amount, and bar plot shows nM ATP per µg/mL proteins ± SEM. Mitochondrial content was measured using flow cytometry and is indicated by MitoTracker Green mean fluorescence ± SEM, and 1 × 10^5^ events were recorded for each replicate. (**D**) MMP and calcium homeostasis. *MMP* was measured with the JC-1 dye and is indicated by the fluorescence ratio between JC-1 aggregates and JC-1 monomers. Dot plot shows mean red/green ratios ± SEM. Cytosolic calcium was measured as the Fura-2 fluorescence ratio F340/380 and is represented as mean ratio ± SEM. Mitochondrial calcium levels were measured using Rhod-2/AM and is presented as mean fluorescence intensity, in relative fluorescent unit ± SEM. (**E**) Cell size was analyzed by assessing area (pixels) of Fura-2/AM-loaded cells. Dot plot shows the number of pixels ± SEM. (**F**) Oxidative stress indicators. Cytosolic reactive oxygen species (ROS) and mitochondrial ROS (superoxide) were measured using flow cytometry and are indicated by DCFDA and MitoSOX mean fluorescence, respectively. Bar plots show mean fluorescence ± SEM, and 2 × 10^4^ events were recorded for each replicate. On the right of each graph, with matching color coding and order, are representative histograms showing fluorescence (x-axis) and cell count (y-axis). Numbers indicate mean fluorescence. Ctl: non-depressed controls cohort (grey); MDD: major depressive disorder cohort (yellow); Ctl 17 and Ctl 18: non-depressed controls (grey); Non-R: non-responder patient (orange); and Mito: mitochondriopathy patient (red). All data were analyzed with paired *t*-test, and significant differences were indicated with * (*p* < 0.05), ** (*p* < 0.005), *** (*p* < 0.0005), and **** (*p* < 0.0001). Mean, SEM, *n*, and *p*-value are compiled in Table S6.

To extend our studies on the bioenergetic properties and mitochondrial function in patient cells of the neural lineage, we implemented the differentiation of NPCs to astrocytes according to the protocol of [42]. To verify successful differentiation, we stained against markers typically expressed in mature astrocytes 30 days after starting the differentiation. Immunofluorescent antibody labeling demonstrated expression of GFAP, ALDH1L1, EAAT1, S100β, and connexin 43 (Figure 3A). Moreover, the presence of ATP-induced wave-like Ca^2+^ signals in the astrocyte cultures indicated that the NPCs differentiated to mature astrocytes (Figure S2). In addition to the Mito and Non-R patients and their direct controls, we generated astrocytes from subjects of our MDD and control cohort [32] to use them as a reference in our study (for cohort information, see Table S5).

#### 2.4.1. Bioenergetic Properties

Basal and maximal respiration, proton leak, and ATP-related and non-mitochondrial oxygen consumption were significantly lower in MDD astrocytes (Figure 3B, Table S6), consistent with findings previously reported in fibroblasts and NPCs and with findings in other cell types described in the literature. In contrast, the basal and maximal respirations of Non-R astrocytes were reduced compared to its controls. Mitochondrial respiration in the Mito patient’s astrocytes did not differ from its control (Figure 3B, Table S6). These data indicate that the OXPHOS in astrocytes of Mito and Non-R patient might be affected in a differential and cell-dependent manner.

As in the other cell types, there was no significant difference in the mitochondrial content in astrocytes. However, the Mito patient’s astrocytes showed significantly decreased ATP concentrations (Figure 3C, Table S6). Representing a further measure of bioenergetic functions, the MMP was decreased in astrocytes from MDD patients and the Mito patient, but significantly increased in the Non-R patient (Figure 3D, Table S6). 

#### 2.4.2. Calcium Homeostasis and Cell Size

Regarding Ca^2+^ homeostasis, the MDD cohort astrocytes displayed slightly, yet significantly reduced, cytosolic Ca^2+^ levels; whereas, there was a marked increase in the Non-R astrocytes and a significant elevation in the Mito astrocytes (Figure 3B, Table S6). Consistent with a decreased MMP, mitochondrial Ca^2+^ levels were lower in the MDD cohort and Mito patient’s cells. In contrast, despite a higher MMP, Non-R astrocytes showed decreased levels of mitochondrial Ca^2+^ as well (Figure 3D, Table S6). Notably, this decrease aligns with lower respiratory rates in this patient’s astrocytes, suggesting a potential causal relationship.

In line with previous observations, cell size was decreased in MDD and Non-R astrocytes. Surprisingly, astrocytes from the Mito patient were found to be larger than those of the corresponding controls (Figure 3E, Table S6). 

#### 2.4.3. Redox Homeostasis

Cellular ROS showed high variability between groups and were altered in opposite directions in the patients’ astrocytes. They were decreased in the Non-R astrocytes but increased in the Mito patient. Mitochondrial superoxide content did not differ (Figure 3F, Table S6).

Taken together, patients’ astrocytes showed contrasting results to fibroblasts and NPCs. Non-R astrocytes were smaller than control astrocytes, and they had lower respiration and mitochondrial Ca^2+^ levels but higher MMP. The Mito patient’s astrocytes had unchanged respiration, lower MMP and mitochondrial Ca^2+^ levels, and higher cytosolic ROS. Both patients’ astrocytes had increased cytosolic Ca^2+^ levels.

### 2.5. Neurons of Patients Show Altered Mitochondrial Membrane Potential and Calcium Homeostasis

#### 2.5.1. Characterization of Neuronal Identity

**Figure 4:**
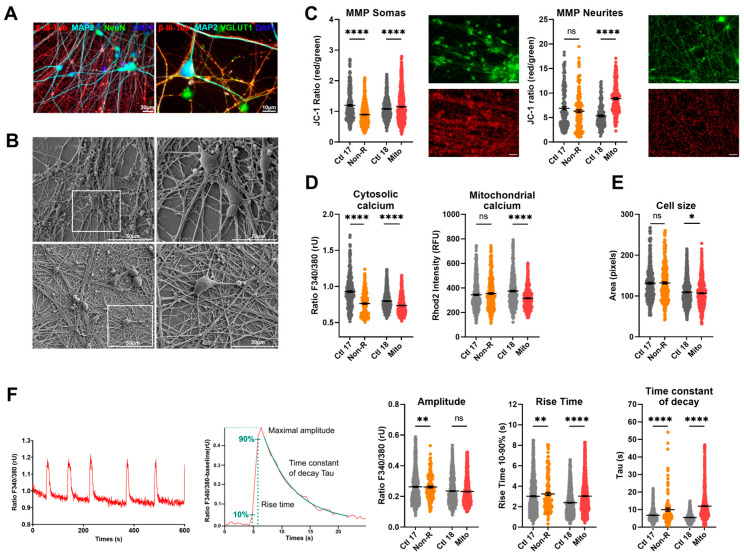
Mitochondrial membrane potential (MMP), calcium homeostasis, and dynamics in iPS-Neurons. (**A**) Neuronal markers. Immunofluorescence stainings on neurons revealed that the induced neurons express typical neuronal cytoskeleton protein MAP2 and β-III-Tubulin and neuronal nuclear marker NeuN. VGLUT1 expression suggests that most of the induced neurons are glutamatergic. Scale bars indicate 20 µm or 10 µm. (**B**) Electron micrographs. Electron micrographs provide high-resolution visualization of neuronal morphology. Scale bars indicate 50 μm (**left**) and 20 μm (**right**). (**C**) MMP in somas and neurites. MMP was measured with the JC-1 dye and is indicated by the fluorescence ratio between JC-1 aggregates and JC-1 monomers. Mitochondria from somas and neurites appeared on different focal planes and were therefore imaged separately. Representative images show red and green JC-1 fluorescence in the relevant structure. Scale bar indicates 20 µm. Dot plot shows mean red/green ratios ± SEM. (**D**) Calcium homeostasis. Cytosolic calcium was measured as the Fura-2 fluorescence ratio F340/380 and is represented as mean ratio ± SEM. Mitochondrial calcium levels were measured using Rhod-2/AM and are presented as mean fluorescence intensity, in relative fluorescent unit ± SEM. (**E**) Cell size was analyzed by assessing area (pixels) of Fura-2/AM-loaded cells. Dot plot shows the number of pixels ± SEM. (**F**) Calcium dynamics. Spontaneous calcium transients were analyzed in Fura-2/AM-loaded cells. Example traces show representative calcium transients in a neuron (**left**) and a baseline-subtracted calcium peak, illustrating maximal amplitude, rise time between 10 and 90% of maximal amplitude, and the exponential fit used to calculate the time constant of decay Tau (**right**). Graphs show the maximum amplitude of the calcium peaks (ratio 340 nm/380 nm ± SEM), the rise time, and the time constant of decay Tau (ms ± SEM). Ctl 17 and Ctl 18: non-depressed controls (grey); Non-R: non-responder patient (orange); and Mito: mitochondriopathy patient (red). All data were analyzed with paired *t*-test, and significant differences were indicated with * (*p* < 0.05), ** (*p* < 0.005), and **** (*p* < 0.0001). Mean, SEM, *n*, and *p*-value are compiled in Table S7.

To investigate functional phenotypes of patient-derived neuronal cells, we differentiated NPCs from cortical-like neurons following the protocol previously used in [32]. We demonstrated successful differentiation by staining against typical neuronal markers (Figure 4A). The neurite network was evidenced with the detection of Microtubule-Associated Protein 2 (MAP2) and β-III-tubulin (β-III-Tub), which are part of the axonal and dendritic cytoskeleton [43,44]. The neuronal marker NeuN stained post-mitotic nuclei and demonstrated a highly pure neuronal culture [45]. The pre-synaptic marker vesicular glutamate transporters 1 (VGLUT1) and the post-synaptic density protein 95 (PSD95) attested the presence of mature synaptic terminals. Additionally, PSD95 serves in the localization of glutamate receptors, and VGLUT1 loads glutamate into synaptic vesicles, making both proteins distinctive markers of glutamatergic neurons [46].

To further illustrate the morphology of the neurons, we produced high-resolution electron micrographs, showing a dense neurite network and protrusions (Figure 4B). Moreover, our recordings demonstrated that the neurons expressed functional glutamate and GABA_A_ receptors (Figure S3).

#### 2.5.2. Mitochondrial Membrane Potential

Mitochondrial function in neurons was first investigated by evaluating JC-1 fluorescence as a measure of MMP. Since mitochondria residing in the somas and in the neurites appeared in different focal planes, we separately imaged JC-1 fluorescence by focusing on the relevant structures, which revealed that MMP can be differentially regulated in these compartments. The MMP of the Non-R neuronal mitochondria was decreased relative to the control. This could indicate mitochondrial dysfunctions and could result in impaired synthesis of macromolecules critical for neuronal functions or altered ability to integrate synaptic inputs [47]. Contrastingly, the Mito patient’s neuronal mitochondria showed increased MMP, especially in the neurites (Figure 4C, Table S7), which could result from a higher respiration rate or a reduced activity of the ATP synthase. Notably, high MMP and OXPHOS rate can provoke reverse electron transfer, leading to a surge in ROS production. 

#### 2.5.3. Calcium Homeostasis

Next, we investigated cellular Ca^2+^ homeostasis in the induced neurons. With our experimental setup, evaluation of Ca^2+^ levels was only possible in the soma, but it was carried out in the cytosol and in mitochondria. Mitochondrial Ca^2+^ levels were significantly decreased in Mito neurons (Figure 4D, Table S7). Ca^2+^ was identified as a key mediator enabling mitochondria to match ATP production to the energy demand resulting from neuronal transmission [48]. Therefore, our results suggest that neurons from the Mito patient might struggle with the dynamic regulation of ATP synthesis.

Intracellular Ca^2+^ was markedly decreased in neurons from both the Non-R and the Mito patients (Figure 4D, Table S7). Given the central role of Ca^2+^ in neuronal function, such a decrease could have profound implications. A decreased baseline concentration of cytosolic Ca^2+^ might result in a lower probability and influence the timing of neurotransmitter release, potentially leading to slower post-synaptic responses. Furthermore, Ca^2+^-dependent short-term plasticity mechanisms could be altered [49]. 

Similar to the MMP, cytosolic Ca^2+^ levels were decreased in the Non-R neurons. In the Mito neurons, decreased cytosolic Ca^2+^ levels were accompanied by decreased mitochondrial Ca^2+^ levels (Figure 4D, Table S7). Cell size was decreased in the Mito patient’s neurons (Figure 4E, Table S7).

### 2.6. Functional Properties and the Activity of Patient-Derived Neurons Are Altered

Electrical activity is a hallmark of neuronal function. High-resolution electron micrographs demonstrated that our patient-derived neurons displayed typical morphology and formed a dense interconnected neurites network, suggesting robust synaptic interactions (Figure 4B). To investigate biophysical properties and functionality of neurons, we performed whole-cell patch-clamp recordings of patient-derived and control neurons. 

#### 2.6.1. Resting Membrane Potential and Capacitance

**Figure 5:**
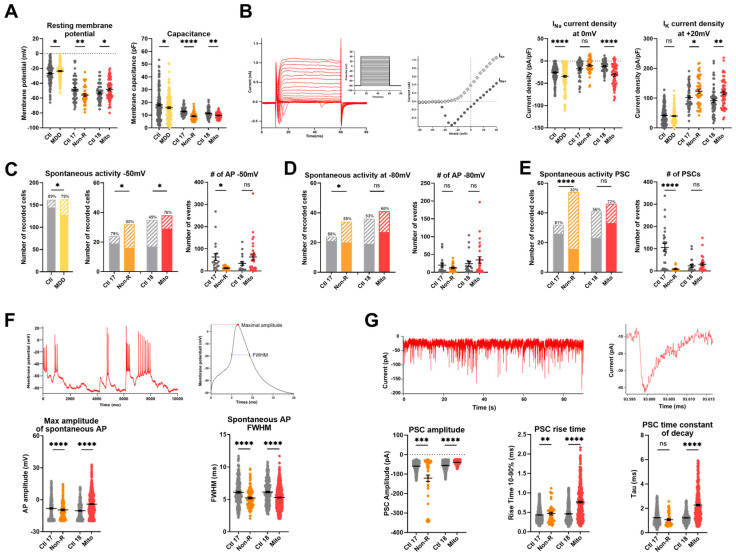
Electrophysiological properties of patient-derived iPS-Neurons. (**A**) Passive biophysical properties. Resting membrane potential (RMP) and capacitance were recorded immediately after reaching the whole-cell configuration. Dot plot shows mean RMP in mV ± SEM and mean capacitance in pF ± SEM. (**B**) Sodium and potassium current densities. Sodium (INa) and potassium (IK) currents were recorded in voltage-clamp mode while holding the membrane potential at −80 mV (Vhold) and depolarizing in steps of 10 mV to provoke the opening of voltage-gated Na^+^ and K^+^ channels. Example traces show depolarizing steps, the evoked Na^+^ and K^+^ current, and the resulting IV curve. Currents measurements were normalized to the membrane capacitance to account for cell size variability (current density, pA/pF). Dot plots show mean INa current density at 0 mV in pA/pF ± SEM and mean IK current density at +20 mV in pA/pF ± SEM. (**C**,**D**) Spontaneous action potentials (APs) were recorded in current-clamp while the membrane potential was held at −50 mV (**C**) or −80 mV (**D**). Spontaneous activity is represented as the proportion of active cells (solid color) and inactive cells (pattern). The percentage of active cells is indicated above each. Dot plots show the mean number of recorded APs ± SEM in case study patients. (**E**) Spontaneous post-synaptic currents (PSCs) were recorded at a holding potential of −80 mV. Left graph shows the proportion of active cells (solid color), inactive cells (pattern), and the percentage of active cells. Right graph shows the mean number of recorded PSCs ± SEM. (**F**) Spontaneous APs analysis. Spontaneous APs at −80 mV were analyzed individually to extract the maximal amplitude and the full width at half maximum (FWHM). Example traces show spontaneous APs (**left**) and a single AP trace illustrating amplitude and FWHM (**right**). Graphs show mean AP amplitude in mV ± SEM and mean FWHM in ms ± SEM. (**G**) Post-synaptic currents (PSCs) analysis. Example traces show spontaneous PSCs (**left**) and one PSC (**right**). Graphs show the maximum amplitude of the PSCs (pA ± SEM), the rise time between 10% and 90% of the maximal amplitude, and the time constant of decay Tau (ms ± SEM). Ctl: non-depressed controls cohort (grey); MDD: major depressive disorder cohort (yellow); Ctl 17 and Ctl 18: non-depressed controls (grey); Non-R: non-responder patient (orange); and Mito: mitochondriopathy patient (red). All data were analyzed with paired *t*-test, except spontaneous activity, which was analyzed with Fisher’s exact test. Significant differences were indicated with * (*p* < 0.05), ** (*p* < 0.005), *** (*p* < 0.0005), and **** (*p* < 0.0001). Mean, SEM, *n*, and *p*-value are compiled in Table S8.

We found that the resting membrane potential (RMP) of the Mito neurons was significantly less negative than in control neurons, as was the case in neurons from the MDD cohort. Interestingly, the Non-R neurons showed a significantly more hyperpolarized RMP (Figure 5A, Table S8). It should be noted that the patient neurons had a more negative RMP than the MDD cohort neurons used in [32]. Optimization of the differentiation protocol may have resulted in a more advanced and mature phenotype of the neurons. 

Consistent with the results of the MMD cohort, the neurons of the case study patients had a lower electrical capacitance, i.e., they were smaller than those of the corresponding controls (Figure 5A, Table S8).

#### 2.6.2. Sodium and Potassium Current Densities

Analysis of the voltage-gated potassium currents revealed that the current density (pA/pF) was significantly higher in neurons of both the Mito and Non-R patients (Figure 5B, Table S8). The voltage-gated sodium channels of Mito and MDD neurons also showed a significantly higher current density, whereas sodium current densities in Non-R neurons did not differ from its control (Figure 5B, Table S8).

#### 2.6.3. Spontaneous Action Potentials

Furthermore, the current-clamp mode was used to adjust the basal membrane potential to approximately −50 mV or −80 mV by current injection and record the potential fluctuations, which occasionally lead to spontaneous action potentials (APs) (Figure 5C,D, Table S8). Again, the patient neurons showed the opposite behavior. The activity of the Mito neurons was significantly increased (higher number of active cells), whereas Non-R neurons were less active (fewer cells showed spontaneous APs, paralleled by a decrease in number of APs in active cells) (Figure 5C,D, Table S8).

The spontaneous APs from Mito neurons were significantly larger and showed shorter full width half maximum (FWHM) time than those of the relevant control (Figure 5F, Table S8). The amplitudes and the FWHM time of Non-R APs were smaller than in the control (Figure 5F, Table S8).

#### 2.6.4. Spontaneous Post-Synaptic Currents

Post-synaptic currents (PSCs) recorded in voltage-clamp experiments (at a holding potential of −80 mV) can be considered a measure of synaptic input. Only 30% of Non-R neurons displayed PSCs, while the proportion was 81% in Ctl 17 (Figure 5E, Table S8). In contrast, a large proportion of Mito neurons (72%) received synaptic input, although there was no significant difference compared with synaptic activity in Ctl 18 (56%) (Figure 5E, Table S8).

Interestingly, the PSCs of Mito neurons differed in various parameters. They were significantly smaller in amplitude, showed an increased rise time_10–90%_, and a prolonged time constant of decay compared to their control (Figure 5G, Table S8). In Non-R neurons, in spite of a small proportion of active cells, PSCs had an increased rise time_10–90%_ and larger amplitude (Figure 5G, Table S8).

#### 2.6.5. Spontaneous Calcium Activity

The electrical activity of the cultured neurons triggered Ca^2+^ signals in the neurons that were analyzed by live-cell imaging using the Ca^2+^-sensitive dye Fura-2/AM. Interestingly, the rise time_10–90%_ and the time constant of decay were increased in the Mito neurons, suggesting prolonged Ca^2+^ signals. However, the rise time was unchanged in the Non-R patient, but the time constant of decay was also increased. The amplitudes of the Ca^2+^ transients were not different between the groups (Figure 4F, Table S7). 

A summarized overview of the alterations reported in this study, as well as corresponding results in the MDD cohort from previous studies [31,32], is provided in Table 2. Additionally, we determined the flux control ratios from mitochondrial respiration data in order to qualitatively compare oxygen consumption levels and to facilitate comparison to the existing and future literature. Net routine, coupling efficiency, routine flux, and leak control ratios in fibroblasts, NPCs, and astrocytes are presented as supplementary figures (Figures S4, S5, and S6, respectively).

## 3. Discussion

Building on previous work on a cohort of MDD patients [31,32], the present study constituted a deeper exploration into the ways in which mitochondria can influence cellular function and potentially contribute to the development of depression. This was achieved by examining closely two atypical patients: an MDD patient who was not responsive to antidepressant treatments (referred to as “Non-R”) and a patient diagnosed with mitochondriopathy (referred to as “Mito”).

We previously reported altered bioenergetics in the MDD cohort, including decreased respiration and MMP [31,32]. Notably, such alterations have also been reported in other neurological disorders including Parkinson’s, Alzheimer’s, and Huntington’s diseases [50]. In the present study, we found significant bioenergetic alterations in both case study patients. 

In the Non-R patient, fibroblasts and NPCs showed a significantly increased mitochondrial respiration. Constant mitochondrial content suggested that higher respiration resulted from increased OXPHOS activity. These results appear unexpected, given that MDD, like other psychiatric and neurodegenerative diseases, is known to involve mitochondrial alterations and reduced bioenergetics [12,13,51]. However, the high OXPHOS activity observed in these cells was not accompanied by an elevated ATP concentration, nor a higher MMP, suggesting an uncoupling of the electron transport chain (ETC) and the ATP synthase. Notably, an increased proton leak was observed. The adenine nucleotide translocator (ANT) is a major catalyst of basal proton leak in mitochondria [52]. It is conceivable that an altered expression or activity of ANT in the Non-R patient’s fibroblasts and NPCs resulted in the higher proton leak observed, which caused a compensatory increase in ETC activity, in a failed attempt to increase the MMP and ATP production. Importantly, the Non-R patient’s increased respiration challenges the mitochondrial hypothesis of MDD, which associates the disorder with decreased respiration. This suggests a revision of the theory to include the possibility of harmful overactivation in mitochondrial function, thus creating a detrimental imbalance. Considering whether a patient with depression exhibits an overactivation or a reduction in mitochondrial functions could have implications for therapeutic responses and pave the way for personalized therapeutic approaches. Moreover, consistently with a hyperactive ETC, mitochondrial ROS were significantly increased in the Non-R fibroblasts. Elevated mitochondrial Ca^2+^ levels in these cells can further increase ROS production and sensitize mitochondria to apoptotic Ca^2+^-induced mPTP openings [53]. 

In contrast to fibroblasts and NPCs, mitochondrial respiration in the astrocytes from the Non-R patient was generally reduced. Ca^2+^ is known to stimulate respiration by activating key enzymes in the TCA cycle. In line with this, respiratory activity and mitochondrial Ca^2+^ levels showed a consistent trend in this patient’s cells: both increased in fibroblasts and NPCs but decreased in astrocytes. One explanation for the metabolic differences in the Non-R patient’s astrocytes could lie in the specificities of astrocytic mitochondrial Ca^2+^ homeostasis. Indeed, astrocytes do not seem to rely on the mitochondrial uniporter (MCU) for Ca^2+^ influx [54], whereas the Na^+^/Ca^2+^/lithium exchanger (NCLX) plays a particularly important role in Ca^2+^ efflux [55,56]. We could hypothesize that the Non-R patient’s cells expressed higher amounts of NCLX, resulting in a particularly enhanced Ca^2+^ efflux from astrocytic mitochondria and, consequently, reduced mitochondrial Ca^2+^ and ETC activity in astrocytes. In contrast, the elevated mitochondrial Ca^2+^ observed in fibroblasts and NPCs from the Non-R patient could result from modulations of MCU expression, a mechanism that would not affect astrocytic mitochondrial Ca^2+^ levels.

In the Mito patient, fibroblasts and NPCs showed significantly reduced oxygen consumption rates. This observation is consistent with a reduced function of the ETC and OXPHOS in MDD, as we reported previously [31,32], and as may be assumed for a patient with mitochondriopathy. However, no known pathogenic disease-associated variants in nuclear genes were detected by exome sequencing of the Mito patient’s fibroblasts nor by mitochondrial genome sequencing [57]. This could indicate that either a sporadic mutation in one of the mitochondrial genes or an environmental factor is responsible for the observed phenotype. Additionally, it is impossible to completely exclude any mitochondrial mutation, as mutated mtDNA could be present only in a subset of the total mtDNA population in a tissue (heteroplasmy) [58]. It is also important to note that the analysis of sequence data should not be regarded as a final assessment of the entirety of all genes. 

The unchanged mitochondrial content in this patient’s cells suggested that lower respiration was due to decreased OXPHOS activity. However, ATP content remained stable, which suggests compensatory glycolytic activity that offsets the cost of running energy-intensive processes [59]. 

In the Mito patient’s fibroblasts, lipid peroxidation was increased, while glutathione antioxidant system was decreased, suggesting compromised antioxidant defenses. Furthermore, in NPCs from this patient, both mitochondrial superoxide and lipid peroxidation were increased, indicating a high oxidative stress. Low respiration, high superoxide, and high MMP together are indicative of reverse electron transfer in these cells [60].

As in the Non-R patient, astrocytes from the Mito patient displayed distinct characteristics. While in the MDD cohort, astrocytic respiration was consistently decreased, astrocytes from the Mito patient exhibited unchanged respiration. Moreover, ATP concentration was significantly decreased, suggesting alterations in other metabolic pathways. Astrocytes predominantly use glycolysis for energy production [61]. Notably, ROS inhibit various glycolytic enzymes including glyceraldehyde 3-phosphate dehydrogenase (GAPDH), and Hyslop and colleagues demonstrated ROS also directly inhibit ATP synthase without decreasing the respiratory chain capacity [62]. Therefore, we hypothesize that the elevated cytosolic ROS detected by DCFDA in this patient’s astrocytes hindered ATP production. Taken together, the absence of an OXPHOS increase despite lower ATP and MMP indicates that astrocytic mitochondria are struggling to meet energy demands efficiently in the Mito patient. 

The tight regulation of cytosolic Ca^2+^ levels is critical for cell viability. The endoplasmic reticulum is the primary Ca^2+^ storage site, and its close interaction with mitochondria at contact sites called mitochondrial-associated membranes (MAMs) facilitates significant Ca^2+^ exchanges [63]. Ca^2+^ levels are also maintained by transporters and pumps, such as the Plasma Membrane Ca^2+^ ATPase (PMCA), which uses ATP to extrude Ca^2+^ out of the cell. In our study, the main change observed over the different patients and different cell types was a rise in cytosolic Ca^2+^ levels. Interestingly, most instances of increased cytosolic Ca^2+^ were accompanied by decreased respiration. Mankad et al. demonstrated that when mitochondrial respiration is altered, even if global ATP concentrations are maintained, PMCA becomes particularly sensitive to small ATP fluctuations [64]. It is therefore plausible that in patients cells exhibiting higher Ca^2+^ levels, perturbations in respiration may have reduced PMCA activity, leading to an intracellular Ca^2+^ rise. Contrastingly, cytosolic Ca^2+^ levels were significantly lower in neurons derived from both patients, which could have implications on neurotransmission if this decrease is also observed in synaptic terminals. Indeed, lower Ca^2+^ at the synapses can result in lower probability of neurotransmitter release and alter Ca^2+^-dependent short-term plasticity mechanisms [49]. While caution is warranted when extending findings from cellular models to clinical symptoms, observed bioenergetic alterations in Non-R, Mito, and MDD cohorts hint at potential links to clinical manifestations. For instance, fatigue in MDD and Mito patients may be related to reduced mitochondrial function, while increased mitochondrial metabolism in Non-R patients could contribute to restlessness and agitation in the context of MDD. Interpretation should be approached with care, acknowledging the challenge of translating cellular observations to clinical complexities.

Cytosolic Ca^2+^ dynamics can impact mitochondrial Ca^2+^ and vice versa. For instance, under stress conditions, mitochondria may release Ca^2+^ into the cytosol as a protective mechanism against apoptosis. Concurrent increased cytosolic Ca^2+^ and decreased mitochondrial Ca^2+^ were observed in astrocytes from both patients. This could reflect cellular stress or result from the unique characteristics of astrocytes concerning mitochondrial Ca^2+^ influx and efflux, as described above. The MMP also impacts mitochondrial Ca^2+^ levels. Here, the mitochondrial Ca^2+^ levels in the analyzed cell lineages only partially follow the observed MMP or cytosolic Ca^2+^ levels. Nonetheless, it is noteworthy that mitochondrial Ca^2+^ levels were altered in all patient-derived cells and that the variations consistently mirrored changes in mitochondrial respiration. This aligns with the activating role of Ca^2+^ on OXPHOS [65] and underscores the central role of mitochondria in the cellular anomalies observed in these patients’ cells. Similarly, in the Mito patient’s neurons, we observed decreased mitochondrial Ca^2+^ despite increased MMP. Considering that ATP concentration is proportional to the rise in mitochondrial Ca^2+^ [48], this observation suggests that the Mito patient’s neurons struggle with the dynamic regulation of ATP synthesis. In the Non-R patient’s neurons, mitochondrial Ca^2+^ was unchanged but the MMP was decreased in somatic mitochondria. Due to technical reasons, we were not able to analyze mitochondrial respiration in neurons. However, assuming that decreased MMP reflects decreased OXPHOS rates and subsequent ATP production in somas, insufficient ATP in the soma could affect the neuron’s ability to integrate these signals effectively, potentially altering its responsiveness to synaptic inputs [47].

Interestingly, we demonstrated that the size of the patient cells was reduced compared to their healthy controls when measured as the sum of fluorescent pixels in fibroblasts, NPCs, and astrocytes and as the electrical capacitance of neurons. This observation is consistent with our earlier findings in NPCs and neurons [32]. The only exception observed was larger astrocytes in the Mito patient, which also differed from other cells by exhibiting elevated cellular ROS. This size increase could therefore result from oxidative stress, as a mechanism to dilute ROS, or as a result of the production of protective proteins to counteract oxidative stress [66]. All other patient astrocytes were consistently smaller than the corresponding controls; so overall, cell size remains a robust symptom/surrogate marker for patient cells. Interestingly, in a recent study where knocking down the mitochondrial protein TSPO resulted in mitochondrial dysfunctions, a decreased cell size was also consistently observed across different cell types [67]. These findings, in conjunction with ours, suggest a relationship between mitochondrial dysfunction and reduced cell size. In general, the neuronal cell size will affect neuronal function since the cell size is affecting the passive biophysical properties (capacitance, membrane resistance, as well as time and length constant) and active propagation mechanisms. Moreover, the spatial and temporal integration of synaptic inputs is also dependent on the size of the soma and the dendritic area. In addition, cell size influences network activity by recruiting distant cell neighbors. Smaller cell size in brain regions related to emotion and mood control has been reported in MDD patients [68]. Taken together, bioenergetic alterations and decreased cell size hold the potential to serve as diagnostic biomarkers for MDD.

We also demonstrated that biophysical properties and function from Mito and Non-R patients’ neurons differ from their corresponding controls. A striking feature of both patient-derived neurons was the markedly increased density of K^+^ currents, which allows for efficient post-AP repolarization of the membrane potential.

In Non-R patient’s neurons, a hyperpolarized RMP with constant Na^+^ current density suggests a bigger hurdle to reach the AP threshold. Consistently, spontaneous activity was significantly lower, i.e., at both −50 and −80 mV, there was a lower frequency of AP in active cells, and the APs observed were smaller and narrower. Moreover, post-synaptic currents (PSCs) were drastically altered with a much smaller fraction of active cells, a drop in event frequency, and the PSCs having extended rise time. Altered synaptic transmission can result in altered synaptic plasticity, a hallmark of MDD [69]. Interestingly, Vadodaria et al. reported altered neurite growth and morphology in neurons derived from serotonin re-uptake inhibitors (SSRIs) non-responder depressed patients. These changes were associated with lowered expression of key Protocadherin alpha genes [70]. Such alterations in the Non-R patient could influence network activity and explain the observed decreases in spontaneous APs and PSCs. Overall, our observations in the Non-R patient’s neurons point to a significant shift in excitability and neuronal transmission. These changes suggest that cortical neurons in the Non-R patient might be less responsive to serotonergic signals due to their hyperpolarization. This could mean that these neurons are less receptive to the increased serotonergic signaling resulting from antidepressant treatment, potentially explaining the Non-R patient’s lack of response to such treatments. 

The Mito patient’s neurons exhibited many similar characteristics to neurons from the MDD cohort patients. Both displayed depolarized RMP, which together with lower capacitance could suggest a compromised energy supply due to mitochondrial dysfunction. This is underscored by the substantial energy demands of maintaining RMP and the pivotal role of mitochondrial biogenesis in axonal growth [71]. Furthermore, the depolarized RMP in Mito and MDD neurons was closer to the AP threshold, and increased Na^+^ currents further promoted depolarization. In the Mito patient, this led to a high proportion of neurons showing spontaneous APs at −50 mV. The APs were taller, yet narrower, consistent with changes in Na^+^ and K^+^ currents. Hyperexcitability is a common feature of mitochondrial disorders, especially in MELAS [72] and MERRF [73]. Supporting this, mice models with induced mitochondrial dysfunction via conditional knock-out of critical mitochondrial proteins showed increased excitability in both glutamatergic neurons [74] and serotonergic neurons [75]. While these studies did not identify the exact cause of hyperexcitability, both suggested a potential disruption in Ca^2+^ homeostasis, with defective Ca^2+^ accumulation in mitochondria following depolarization. This aligns with the decreased mitochondrial Ca^2+^ levels observed in the Mito patient’s neurons. 

Additionally, spontaneous PSCs had significantly lower amplitude and longer rise and decay times, potentially indicating changes in neurotransmitter release. There is increasing evidence linking mitochondrial dysfunction to synaptic transmission failures in Alzheimer’s disease. Notably, patients with early-stage Alzheimer’s disease show synaptic mitochondria issues even before significant synaptic damage occurs [76]. Considering this, it is plausible that the synaptic transmission alterations observed in the Mito patient stem from impairments of neuronal mitochondria.

## 4. Limitations

Interindividual differences could be exacerbated by the differences in the age of the patient/control pairs. Supporting this assumption, we observed a positive correlation of various functional parameters, such as maximal oxygen consumption and spare respiratory capacity, with age in the non-depressed control cohort (*n* = 16, Figure S7). Furthermore, it has been widely demonstrated that there is an age-associated increase in steady-state concentrations of lipid peroxidation products [41] and oxidative DNA damage (OH8dG; [40]). Future studies should aim to include patients closer in age or carefully consider age as a variable when examining mitochondrial dysfunction.

In view of the interindividual variability in direct comparison of two subjects, we aimed to relate the data of the presented case studies to the data obtained from a larger cohort of MDD patients and controls whenever possible. However, we recognize that our results reflect the observations of independent individuals and cannot be interpreted as generally applicable interpretations of MDs and TRD. Future studies investigating certain alterations reported here and validating our findings in larger, more diverse cohorts are crucial. 

The MDD patients from our cohort study were considered no longer depressed at the time of the biopsies, as assessed with the Hamilton Depression Rating scale. Indeed, the patients’ scores were 10.8 ± 1.9, which is recognized as sub-threshold, mild depression [77]. Therefore, our study does not address depressive states, but rather trait markers.

No clear diagnosis on the Mito patient was available, although several clinical parameters, as well as our initial respiratory measurements, indicated a mitochondria-associated disease. Future research involving more precisely defined mitochondrial pathologies would provide further insights. 

Limitations exist in sequencing data analysis, for example, in the detection of low-grade mosaics, of repeat expansions, of balanced changes (translocations and inversions), and in the calling accuracy of larger Indels. Furthermore, in exome sequencing, variants in non-enriched regions (untranslated regions, introns, promoter, and enhancer regions) cannot be detected. In general, accuracy is limited for all variant types in regions with high sequence homology or low complexity or with other technical challenges.

Reprogramming of fibroblasts may affect the expression of disease-associated epigenetic memories. However, we have already shown that functional mitochondrial phenotypes are transmitted (at least partially) to the iPS-derived lineages [32,78]. Moreover, analyzing epigenetic markers, such as the methylome, might provide valuable insight on this point. A different approach to generate neurons directly from fibroblasts [79], thus avoiding reprogramming, would ensure the retention of epigenetic memories. 

While our cellular models have provided valuable insights into mitochondrial dysfunction in MDD pathophysiology, we recognize the inherent complexity of in vivo systems. Extrapolating findings from isolated cellular contexts to the intricacies of whole organisms involves inherent limitations. Future studies in in vivo models are essential for a comprehensive understanding of the broader physiological implications of our observations.

## 5. Materials and Methods

### 5.1. Generation of Control and MDD Patient iPSCs from Fibroblasts

Skin biopsies were conducted by the Department of Dermatology, Regensburg University Hospital, Germany. The study was approved by University of Regensburg’s ethics committee (ref: 13-101-0271), and all participants provided written informed consent. Human fibroblasts were obtained and cultivated as previously described [31]. Fibroblasts from healthy age- and sex-matched controls were also obtained. The non-responder patient is hereafter referred to as “Non-R” and the corresponding control as “Ctl 17”. The mitochondriopathy patient is referred to as “Mito” and the corresponding control as “Ctl 18”. 

iPSCs were generated using the episomal protocol described by [80]. Briefly, 5 × 10^5^ fibroblasts were electroporated with 600 ng of each of the episomal vectors pCBX-EBNS, pCE-hsk, pCE-hUL, pCE-hOCT3/4, and pCE-mp53DD using the Amaxa Nucleofactor (Lonza, Basel, Switzerland). The cells were then cultured in TeSR-E7 medium on Matrigel-coated dishes (Corning, Tewksbury, MA, USA) until colonies appeared. iPSC colonies were manually picked and cultured on Matrigel with mTeSR1 medium.

### 5.2. iPSC Differentiation to NPCs and Neuron Differentiation

iPSCs to neural progenitor cells (NPCs) differentiation was carried out following a monolayer culture method developed by Yan et al. [80]. Small iPSC colonies were plated on Matrigel-coated plates in Neural Induction Medium (Neurobasal Medium, 2% Neural Induction Supplement, 0.5% Penicillin/Streptomycin). On day 7, the differentiating cells were dissociated using Accutase (Life Technologies, Carlsbad, CA, USA), passed through a 50 μm strainer and further cultured in Neural Expansion Medium (Neurobasal/Advanced DMED F12, 2% Neural Induction Supplement, 0.5% Penicillin/Streptomycin) on Geltrex-coated plates. After 5 passages, a pure culture of mature NPCs was obtained.

For neuronal differentiation, 3.5 × 10^4^ NPCs from passages 5 to 12 were placed onto polymer imaging μ-dishes (Ibidi, Gräfelfing, Germany ) coated with 20% poly-L-ornithine in PBS and 43 μg/mL laminin in DMEM/F12, both overnight at 37 °C. On the next day, medium was changed to Neurobasal medium with 1% B27, 0.5% GlutaMax, 0.5% non-essential amino acids, 0.5% Culture One (Thermo Fisher Scientific, Carlsbad, CA, USA), 200 nM ascorbic acid (Carl Roth, Karlsruhe, Germany), 20 ng/mL BDNF and GDNF (PeproTech, Rocky Hill, CT, USA), 1 mM dibutyryl-cAMP (Stemcell, Vancouver, BC, Canada), 4 μg/mL laminin (Sigma, St. Louis, MO, USA), 50 U/mL penicillin, and 50 μg/mL streptomycin (Thermo Fisher Scientific, Carlsbad, CA, USA). Cells were differentiated for 21 days, with half of the medium changed every 3 to 4 days. In order to remove proliferating cells, the cultures were treated with the mitotic inhibitor cytarabine (Biomol, Hamburg, Germany) at 1 μM from day 5 to day 6 or 7. Live-cell imaging experiments were performed on days 20 and 21 of differentiation, and patch-clamp experiments were performed between day 19 and day 21 of differentiation. No influence of differentiation day was observed.

### 5.3. Astrocyte Differentiation

NPCs were differentiated into astrocytes following a protocol adapted from [42]. A total of 3 × 10^4^ carefully dissociated NPCs were seeded on Matrigel-coated plates in astrocyte media containing 2% FBS, 1% astrocytes growth supplement, and 1% penicillin/streptomycin solution (ScienCell, Carslbad, CA, USA). Upon confluence, cells were detached, and 3 × 10^4^ cells were plated in new wells. After 30 days of differentiation, identity and maturity of the astrocytes were confirmed with immunostainings of typical astrocytes markers (GFAP, S100β, connexin 43, EAAT1, ALDH1L1). Astrocytes were grown on Matrigel-coated plates, and all experiments were performed from day 30 to day 60 of differentiation. 

### 5.4. Analysis of Mitochondrial Respiration

Mitochondrial respiration was analyzed using Seahorse XFp Flux analyzer with a Seahorse XFp Mito Stress Test Kit (Agilent Technologies, Santa Clara, CA, USA) according to manufacturer’s recommendations. The day prior to the assay, 3 × 10^4^ (fibroblasts, astrocytes) or 8 × 10^4^ (NPCs) cells were grown in XFp 8-well miniplates with appropriate coating. Oxygen consumption rates (OCRs) were measured with sequential injection of 1 µM oligomycin, 2 µM (fibroblasts, astrocytes) or 1 µM FCCP (NPCs), and each 0.5 µM rotenone/antimycin A (Biomol, Hamburg, Germany). Respiration rates were normalized to the number of cells by counting DAPI-stained nuclei with ImageJ using a macro involving a thresholding step, segmenting the thresholded image into singular nuclei and counting the nuclei. Respiration measurements were performed on at least three biological replicates.

### 5.5. Immunofluorescence

Immunofluorescence stainings were carried out as described in [32]. Primary antibodies used were anti-β-III-tubulin (mouse; 1:2000, G7121, Promega, Madison, WI, USA), anti-MAP2 (chicken; 1:5000, ab5392, Abcam), anti-VGLUT1 (rabbit; 1:500, ab180188, Abcam, Cambridge, UK), anti-NeuN (rabbit; 1:500, ab177487, Abcam, Cambridge, UK) anti-ALDH1L1 (rabbit; 1:500, ab87117, Abcam), anti-EAAT1 (rabbit; 1:200, ab416-1001, Abcam, Cambridge, UK) anti-GFAP (mouse; 1:400, C53893, Sigma-Aldrich, St. Louis, MO, USA), and anti-connexin 43 (mouse; 1:500, 14-4759-82, Invitrogen, Carlsbad, CA, USA). Secondary antibodies used were anti-mouse Cy3 (1:1000, Thermo Fisher Scientific), anti-rabbit 488, and anti-chicken Cy5 (1:1000, Abcam). Nuclei were stained with DAPI (1:1000, Sigma-Aldrich), and coverslips were mounted using Dako Fluorescing Mounting Medium.

### 5.6. Luminescent Assay for ATP Content

For the quantification of cellular ATP content, 1 × 10^5^ fibroblasts and astrocytes or 1 × 10^6^ NPCs were pelleted and stored at −20 °C. ATP content was measured using CellTiter-Glo^®^Cell Viability Kit (Promega) according to manufacturer’s instructions. Cell pellets were resuspended in 500 µL PBS, heated at 100 °C for 2 min, and kept on ice. Triplicates of 50 µL of sample or standard were applied to a 96-well plate with 50 µL of CellTiter-Glo^®^Reagent. Luminescence was measured at an integration time of 1 s. The RLU was used to calculate the ATP content using a 1 nM to 10 µM standard curve. Concentrations were normalized to µg/mL protein using a BCA assay (Thermo Fisher Scientific). ATP was measured in three to four biological replicates.

### 5.7. Luminescent Assay for NAD/NADH Ratio

To measure substrate availability, 5 × 10^2^ fibroblasts or 5 × 10^3^ NPCs were seeded in duplicates in 96-well plates. NAD/NADH ratio was measured with NAD/NADH-Glo™ assay (Promega) according to manufacturer’s instructions. Cells from three to five biological replicates were used. Briefly, cells were lysed in a base solution containing dodecyltrimethylammonium bromide. For NAD^+^ detection, 0.4 N HCl was added to the lysis solution, and the plate was heated at 60 °C for 15 min. Then, NAD^+^ and NADH wells were buffered with Trizma^®^ and HCl/Trizma^®^ solutions, respectively. NAD/NADH-Glo^TM^ detection reagent was added and incubated for 30 min before recording luminescence.

### 5.8. Luminescent Assay for GSH/GSSG Ratio

Reduced and oxidized forms of glutathione were measured in three biological replicates with the GSH/GSSG-Glo™ assay (Promega) according to manufacturer’s instructions. A total of 5 × 10^2^ fibroblasts were seeded in duplicates in 96-well plates. The next day, cells were lysed with either total or oxidized glutathione lysis reagent for 5 min on a plate shaker at room temperature (RT). Luciferin generation and detection reagents were subsequently added and incubated at RT for 30 and 15 min, respectively, before recording luminescence.

### 5.9. Lipid Peroxidation ELISA

The levels of the lipid peroxidation marker 8-isoprostane were measured in fibroblasts and NPC culture supernatants, using the 8-isoprostane ELISA Kit (Cayman Chemicals, Hamburg, Germany) according to manufacturer’s instructions. Fibroblasts or NPCs were plated in serum-free medium. Upon confluence, cells were detached and counted, while supernatants were collected. Supernatants were frozen with 1:1000 antioxidant butylated hydroxytoluene to avoid lipid degradation. Supernatants were measured in triplicates in the ELISA plate, where three and two biological replicates were measured in fibroblasts and NPCs, respectively. The 8-isoprostane concentrations were calculated using a standard curve and normalized to the number of cells.

### 5.10. Imaging of Mitochondrial Membrane Potential (JC-1) Cytosolic Ca^2+^ (Fura-2/AM) and Mitochondrial Ca^2+^ (Rhod-2/AM)

Live-cell imaging experiments were performed using Zeiss Axio Observer Z.1 microscope equipped with a Fluar 40/1.3 objective lens (Zeiss, Jena, Germany). All recordings were performed with an AxioCam MRm CCD camera (Zeiss) and a 40× oil immersion objective. The Lambda DG-4 high-speed wavelength switcher (Sutter Instrument, Novato, CA, USA) was used for illumination and image acquisition, and the microscope was controlled using ZEN 2012 imaging software (version 2.0.0.0). For the analysis, regions of interest were manually drawn around cells using ImageJ (version 2.9.2) [81,82]. Macros were used for background subtraction and, where applicable, to calculate ratios in order to ensure the repeatability of the analysis. Additionally, cell size was measured in Fura-2/AM-loaded cells. Experiments were repeated in three to five independent samples.

The day before experiments, 1.5 × 10^5^ fibroblasts, 1.5 × 10^5^ astrocytes, or 2 × 10^6^ NPCs were plated on uncoated, Matrigel- or Geltrex-coated glass coverslips, respectively. For neurons, 3.5 × 10^4^ NPCs were plated and differentiated for 21 days on PLO/laminin-coated Ibidi dishes. For MMP measurement, cells were loaded with JC-1 at a concentration of 300 nM in fibroblasts and astrocytes and 1 μM in NPCs and neurons. For calcium measurements, cells were loaded with 2 μM Fura-2/AM and 2 μM Rhod-2/AM in OptiMem. Cells were incubated at 37 °C for 30 min. JC-1 fluorescence was measured at 537/42 nm (green) and 620/60 nm (red) after excitation at 480/36 nm. In neurons, neurites and somas appeared on different focus planes and were imaged separately. 

Fura-2 fluorescence was measured at 510 nm after excitation at 340 or 380 nm. Rhod-2 fluorescence was measured at 576 nm after excitation at 556 nm. In neurons, in addition to basal cytosolic Ca^2+^ measurements, spontaneous Ca^2+^ peaks were recorded over 20 min with 2 Hz frequency using the Fluar 20X/0.75 objective lens. Spikes were analyzed with IGOR Pro software (version 9.05; WaveMetrics, Lake Oswego, OR, USA).

### 5.11. Flow Cytometry (MitoTracker Green, DCFDA, MitoSOX)

Flow cytometry was used to detect ROS and mitochondrial content in fibroblasts, NPCs, and astrocytes using specific fluorescent dye. DCFDA (2′,7′-dichlorofluorescein diacetate) (10 µM, 20 min) was used to detect cytosolic hydrogen peroxide and peroxyl radicals, mitochondrial superoxide was detected with MitoSOX (5 µM, 30 min), and mitochondrial mass was investigated using MitoTracker Green (1 µM, 1 h). For MitoTracker Green staining, cyclosporine A 500 µg/mL was used to prevent mitochondria depolarization. A total of 2 × 10^4^ events were recorded for fibroblasts and astrocytes, and 1 × 10^5^ events were recorded for NPCs. Samples were acquired with the FACS Celesta^TM^ Cell Analyzer and analyzed using FlowJo software (V10.8, Tree Star). Flow cytometry measurements were performed on three to nine biological replicates, depending on the variability of the data acquired.

### 5.12. Electrophysiology

Whole-cell patch-clamp recordings were performed on induced neurons during their 4th week of differentiation. The extracellular solution was composed of 140 mM NaCl, 5 mM KCl, 2 mM CaCl_2_, 1 mM MgCl_2_, 10 mM HEPES, and 5 mM Glucose, pH 7.3. Micropipettes were made of borosilicate glass (Science Products, Hofheim, Germany) by means of a horizontal pipette puller (Zeitz Instruments, Munich, Germany) and were fire-polished to obtain a series resistance of 3–5 MΩ. Micropipettes were filled with intracellular solution (140 mM KCl, 1 mM MgCl_2_, 0.1 mM CaCl_2_, 5 mM EGTA, 10 mM HEPES). Recordings were made using an HEKA Electronic EPC-10 amplifier (HEKA Electronic, Reutlingen, Germany). The liquid–liquid junction potential was calculated to be 4 mV (LJP calculator of pClamp software suite, version 11, Axon Instruments, San Jose, CA, USA) but not corrected. The series resistance was assessed but not compensated. The resting membrane potential (RMP) and capacitance were recorded directly after reaching the whole-cell configuration. For voltage-clamp recordings, membrane potential was held at −80 mV and depolarized in steps of 10 mV to evoke voltage-activated Na^+^- and K^+^-channels. Spontaneous post-synaptic currents were recorded while holding the membrane potential at −80 mV. In current-clamp mode, manually adjusted currents were injected to hyperpolarize the membrane potential to about −80 mV or −50 mV and to record spontaneous action potentials. All patch-clamp recordings were carried out at room temperature. Data were analyzed using Patchmaster Next (version 1.3; HEKA Electronic). Cells with RMP of 0 mV and above were excluded from the analysis. Recordings were performed on five (Ctl 18), seven (Non-R, Mito), and nine (Ctl 17) independent neuronal differentiations. The number of neurons (*n*) included for each parameter after outlier exclusion is indicated in Table S8.

### 5.13. Statistical Analysis

Graphical depiction and statistical analysis were conducted with GraphPad Prism 9.5.1 (GraphPad Software). For all experiments, the means of two to three technical replicates were calculated, and biological replicates were averaged. The number of biological replicates is indicated for each experiment in the corresponding section of the methods. A technical replicate refers to the same experimental procedure being repeated multiple times on the same sample. Biological replicates refer to independent samples. Measurements were conducted pairwise allowing direct comparison. Statistical outliers were detected and eliminated using ROUT-Method, except for the number of events in spontaneous activity experiments (post-synaptic currents and action potentials). Results of spontaneous activity were compared using Fisher’s exact test. All results showed variance homogeneity and were compared using paired Student’s *t*-test. Our analysis utilized a significance level of α = 0.05 to control the Type I error rate. Results were presented as mean ± SEM. *p*-value limit for statistical significance was set to ≤0.05. Tables S1, S4, S6, S7, and S8 present mean ± SEM, *n*, and *p*-value for each comparison.

## 6. Conclusions

In the non-responder patient (Non-R), fibroblasts and NPCs exhibited markedly increased respiration but constant MMP, suggesting proton leak, potentially via the adenine nucleoside transporter. The high OXPHOS activity could result from the starkly elevated mitochondrial calcium levels and led to high mitochondrial ROS. In neurons from this patient, evidence suggested compromised synaptic transmission. MMP was decreased in the soma, basal calcium was lowered, calcium peaks were extended, and spontaneous post-synaptic currents were markedly reduced. The Non-R patient’s neurons also displayed decreased excitability, attributed to the hyperpolarization of the resting membrane potential (RMP), leading to fewer spontaneous action potentials. This altered network activity might be linked to altered neurite growth. Overall, these changes might make the patient’s cortical neurons less responsive to serotonergic neurons innervating the cortex. This could potentially explain the patient’s non-responsiveness to serotonin-increasing antidepressant treatments. 

Interestingly, cells from this Non-R patient often presented a contrasting functional phenotype, which starkly deviated from the expected, thus highlighting deeper layers of complexity in the disease and pointing to the involvement of different mechanisms in the etiology of depression. The bioenergetics hypothesis of MDD seems to be more nuanced than a strict decrease in function and to also encompass an overactivation of certain mitochondrial functions, thereby creating a detrimental imbalance. Further studies focusing on increased mitochondrial metabolism in depressed patients would be highly valuable for a more comprehensive understanding of MDD. Moreover, determining the direction of mitochondrial function alterations might inform the development of targeted therapies and improve therapy responses in atypical MDD patients. 

In the mitochondriopathy patient (Mito), generally impaired bioenergetic functions were evident, characterized by a decreased respiration and depolarized MMP, whereas oxidative stress indicators were high. In NPCs, the combination of high MMP, low respiration, and elevated ROS suggested reverse electron transport, potentially impacting cell function and neuronal differentiation. Neurons from the Mito patient had high MMP and exhibited hyperexcitability, linked to a depolarized RMP and increased sodium current density. The decreased mitochondrial calcium implied that Mito neurons struggled adjusting to the energy demands of neurotransmission and coincided with reduced cytosolic calcium and prolonged calcium peak durations, leading to diminished and slower synaptic currents. 

It is important to highlight that cells derived from this Mito patient mirrored the (dys)function observed in the MDD cohort cells in many cellular and mitochondrial functional parameters, supporting the hypothesis that mitochondria play a crucial role in the pathophysiology of depression. Moreover, valuable insights into prospective research directions emerge from these findings, particularly focusing on the oxidative stress/antioxidant dynamics and the kinetics of spontaneous neuronal activity in an MDD patient cohort.

Finally, cells from both patients were overall smaller in size. Given the role of mitochondria in determining optimal cell size, this could be a marker for mitochondrial dysfunction and inform future diagnostic and therapeutic approaches.

Overall, the present study emphasizes the importance of closely investigating atypical patients to gain a more comprehensive understanding of the multifaceted pathophysiology of MDD, which could benefit the future development of personalized treatment strategies.

## Figures and Tables

**Table 1:** Patient information.

	Non-Responder Patient		
**Age, Sex**	**43, Male**		
Clinical findings	Symptoms		Ratings
	- Depressive mood - Guilt feelings - Suicide ideation - Insomnia and disturbed sleep - Severe somatic symptoms of anxiety - Loss of appetite and weight loss - Loss of interest in any activity		- Hamilton Depression Rating score at the beginning of the in-patient stay at the clinic: 34 (very severe) - Hamilton Depression Rating score after 2 months and several treatment attempts: 22 (severe)
Treatments	Antidepressants - Duloxetine - Venlafaxine - Venlafaxine + lithium Others - Enalapril (hypertension)		
	**Mitochondriopathy Patient**		
**Age, Sex**	**18, Female**		
Clinical findings	Symptoms		Excluded pathologies
	- Fatigue and hypersomnia - Migraines - Pain in wrist, back, shoulders, feet, fingers, elbows, and hips - Numbness in arms and legs - Paresthesia in arms and legs - Loss of visual acuity: myopie and hypermetropie - Gastrointestinal symptoms: nausea, vomiting, and diarrhea		- Inflammatory bowel disease - Immune-related diseases - Thyroid disorders - Ankylosing spondylitis - Rheumatoid arthritis - Osteoporosis - Borreliosis - Optic neuritis - Sacroiliitis - Orthopedic problems - Sarcoma - Neuroblastoma
Blood tests relevant to diagnosis	Measured parameters	Alterations	Descriptions
	LDH2	Decreased	Enzymes involved in lactate metabolism
	LDH4	Increased	
	Citrate	Starkly decreased	Enzymes involved in tricarboxylic acid cycle
	Cis-aconitate	Starkly decreased	
	Isocitrate	Starkly decreased	
	Pyruvate kinase M2	Increased	Enzyme catalyzing the last step of glycolysis
	Vitamin D3	Decreased	Regulator of mitochondrial respiration and oxidative stress
	Coenzyme Q10	Decreased	Electron carrier in the electron transport chain
Urine test	5-Hydroxyindolacetatic acid	Starkly decreased	Serotonin metabolite, low levels linked with depression

**Table 2:** Overview of results. Main findings are summarized in this table. Arrows represent increases (↗) and decreases (↘), and asterisks reflect the significance of the difference. Equal signs (=) represent an unchanged parameter. Significant differences were indicated with * (*p* < 0.05), ** (*p* < 0.005), *** (*p* < 0.0005), and **** (*p* < 0.0001).

	Non-R			Mito			MDD Cohort		
	Fibroblasts	NPCs	Astrocytes	Fibroblasts	NPCs	Astrocytes	Fibroblasts	NPCs	Astrocytes
**Respiration**	↗	↗	↘	↘	↘	=	↘	↘	↘
**ATP**	=	=	=	=	=	↘ *	↘ *	=	=
**MMP**	=	=	↗ ****	↘ ****	↗ ****	↘ *	↘ ****	=	↘ ****
**Cytosolic Ca^2+^**	=	=	↗ ****	=	↗ ***	↗ *	=	↗ *	↘ ***
**Mitochondrial Ca^2+^**	↗ ****	↗ ****	↘ ****	=	= (↘)	↘ ****			↘ ****
**Cell size**	↘ ****	↘****	↘ ****	↘ *	↘ ****	↗ **	↘ ****	↘****	↘ ****
	*Neurons*								
**MMP somas**	↘ ****			↗ ****					
**MMP neurites**	=			↗ ****					
**Cytosolic Ca^2+^**	↘ ****			↘ ****					
**Mitochondrial Ca^2+^**	=			↘ ****					
**RMP**	↗ ** (hyperpolarized)			↘ * (depolarized)			↘ * (depolarized)		
**Capacitance**	↘ ****			↘ **			↘ *		
**I_Na+ 0 mV_ curr. density**	=			↘ ****			↘ ****		
**I_K+ 20 mV_ curr. density**	↗ *			↗ **			=		
**Spontaneous activity**	**−50 mV**	**−80 mV**	**PSCs**	**−50 m**V	**−80 mV**	**PSCs**	**−50 mV**		
	↘*	↘*	↘****	↗ *	=	=	↘*		
**Spont. AP amplitude**	↘ ****			↗ ****					
**Spont. AP FWHM**	↘ ****			↘ ****					
**PSC amplitude**	↗ ***			↘ ****					
**PSC rise time**	↗ **			↗ ****					
**PSC decay time (Tau)**	=			↗ ****					

## Data Availability

Data are contained within the article and Supplementary Materials.

## Supplementary Materials

The following supporting information can be downloaded at: https://www.mdpi.com/article/10.3390/ijms25020963/s1.
