# Supplementary material for: Mitochondrial and Cellular Function in Fibroblasts, Induced Neurons, and Astrocytes Derived from Case Study Patients: Insights into Major Depression as a Mitochondria-Associated Disease
Source: Int J Mol Sci. 2024 Jan 12;25(2):963. doi: 10.3390/ijms25020963 (PMC10815943; doi:10.3390/ijms25020963)
Supplement: Supplementary file 1 [file ijms-25-00963-s001.zip › ijms-2771048-supplementary.pdf]

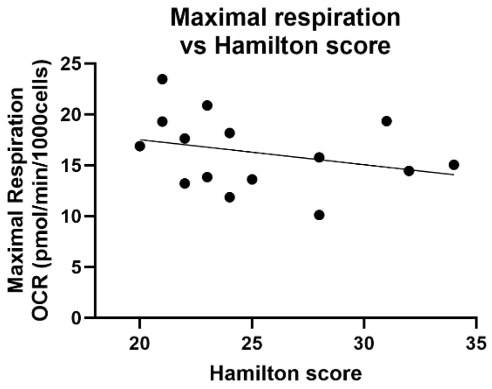

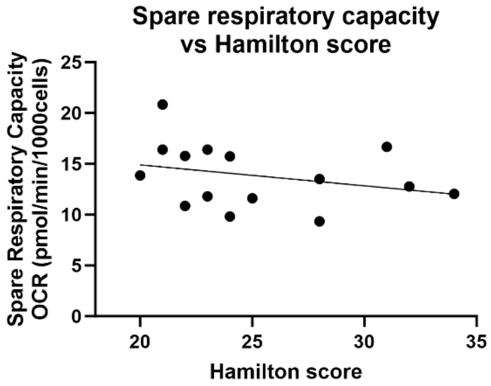

Figure S1. Significant correlations between depression severity and biological findings. Graphs represent Spearmann correlation between respiratory data in the patients with MDD cohort (n = 16) and a simple linear regression. P-values are presented in Table S2.

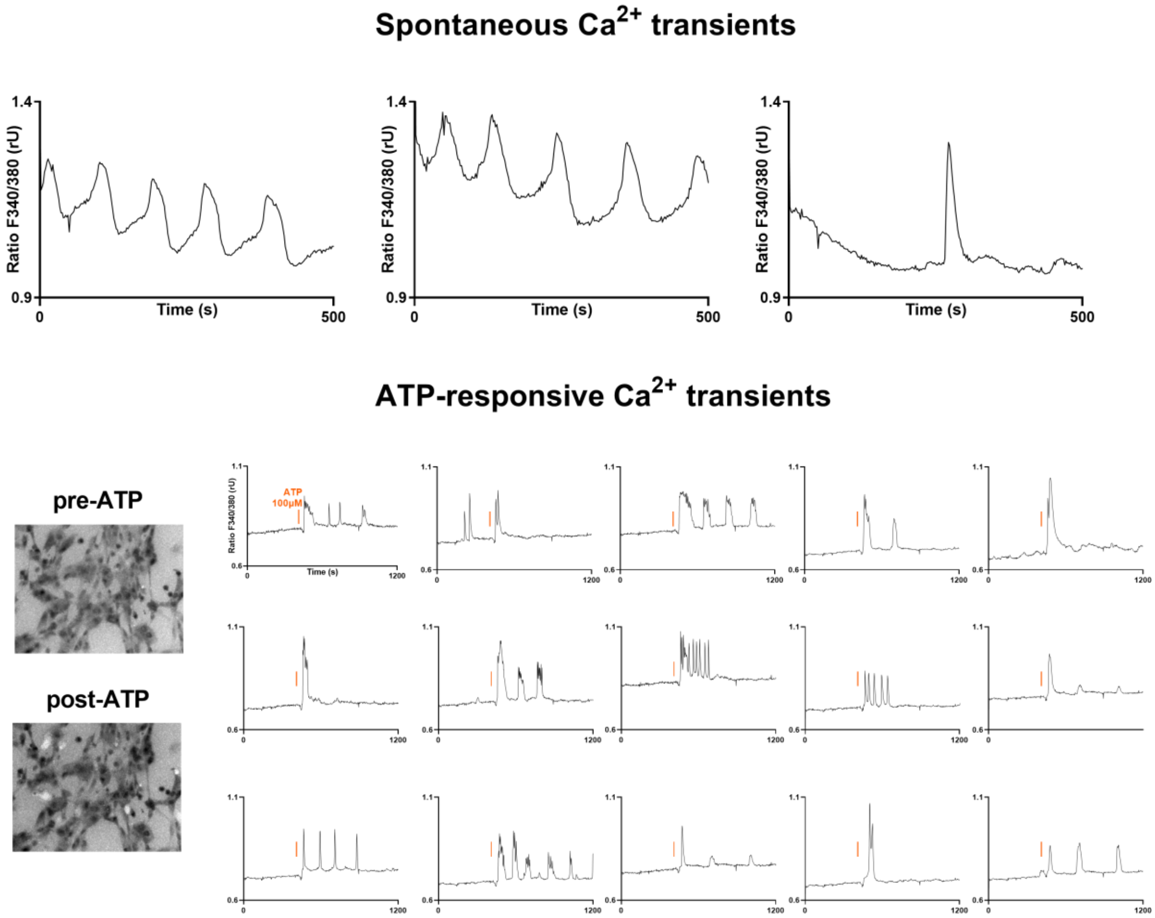

Figure S2. Calcium transients in astrocytes. Example spontaneous calcium transients in astrocytes loaded with Fura-2/AM. Curves show Fura ratio F340/380 nm in relative units (up). Example ATP-responsive calcium transients in in astrocytes loaded with Fura-2/AM. ATP was added to a final concentration of 100µM. On the left, example Fura ratio images showing brighter cells post-ATP. Curves show Fura ratio F340/380 nm in relative units (down)

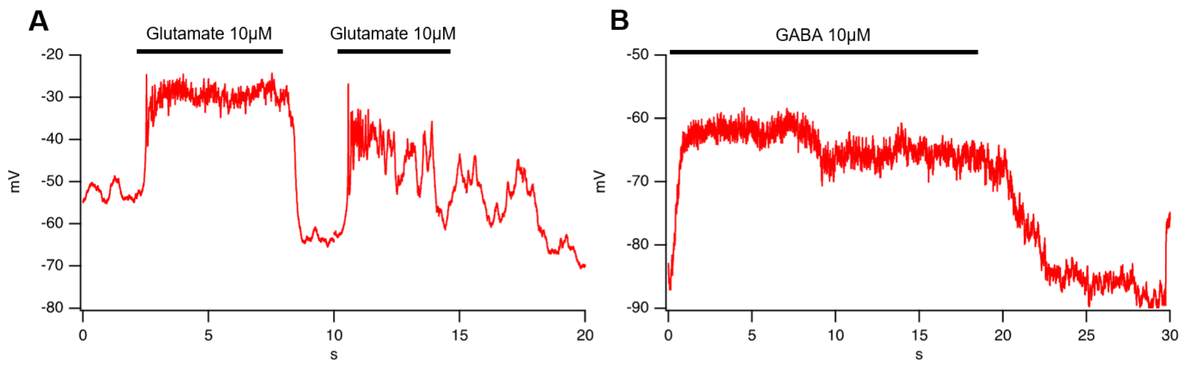

Figure S3. Glutamate and GABA response in current-clamp. Neurons were recorded in whole-cell patch clamp, in current clamp mode without injection of current. Membrane potential was measured while applying 10µM glutamate **(A)** or γ-Aminobutyric acid (GABA) **(B)** using a micro-perfusion system. Bars represent application duration. Note that the direction of GABA response results from a symmetrical chloride concentration in the bath and pipette solutions.

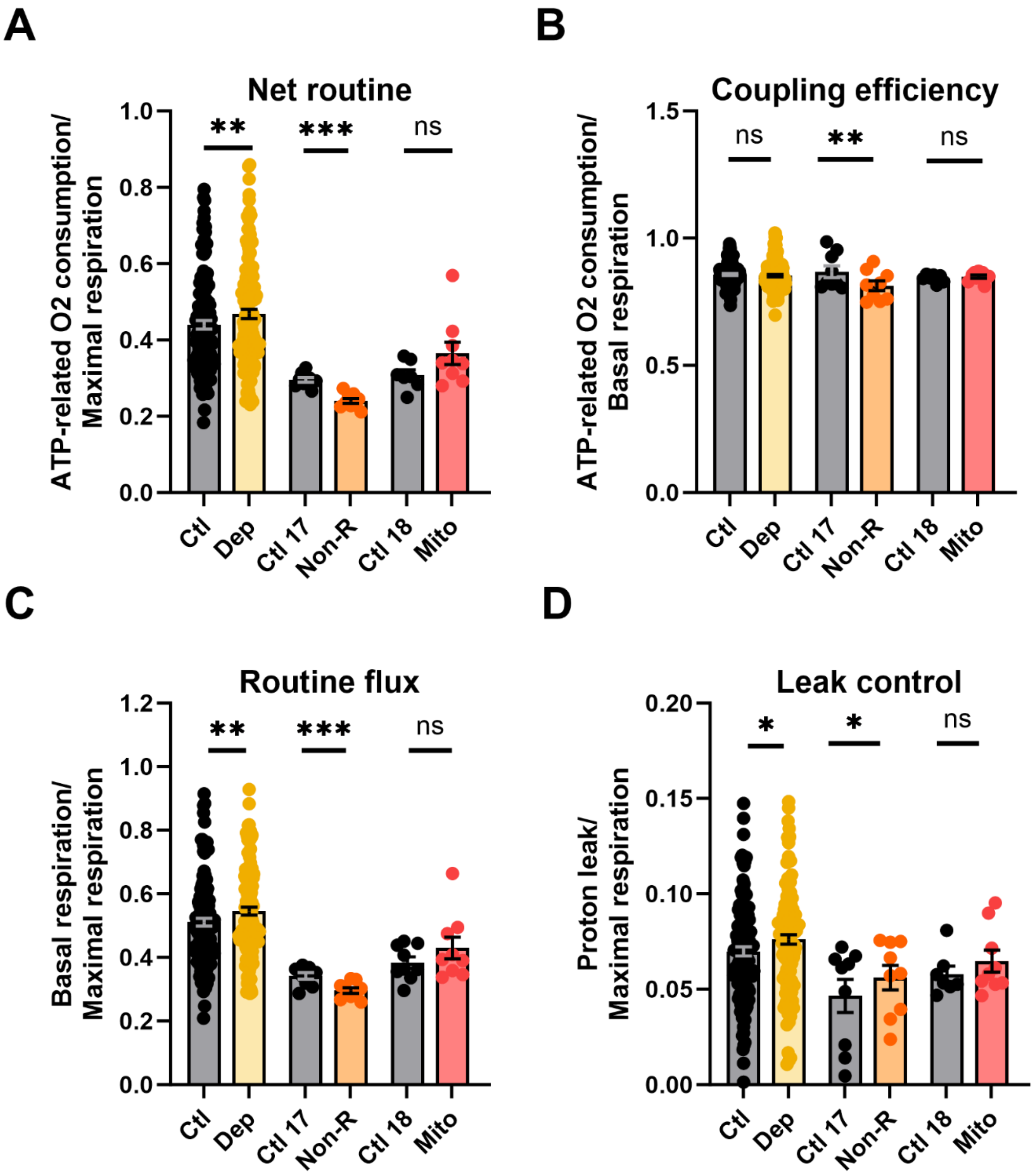

Figure S4. Mitochondrial flux ratios in fibroblasts. (A) Net routine is the ratio between ATP-related O_2 consumption and maximal respiration **(B) Coupling efficiency** is the ration between ATP-related O_2 consumption and basal respiration **(C) Routine flux** is the ratio between basal and maximal respiration **(D) Leak control** is the ratio between proton leak and maximal respiration. Dot plots show the respective ratios ± SEM Ctl: non-depressed controls cohort; MDD: major depressive disorder cohort; Ctl 17 and Ctl 18: non-depressed controls; Non-R: non-responder patient; Mito: mitochondriopathy patient. All data were analyzed with paired t-test, significant differences were indicated with * ($p<0.05$), ** ($p<0.005$), *** ($p<0.0005$)

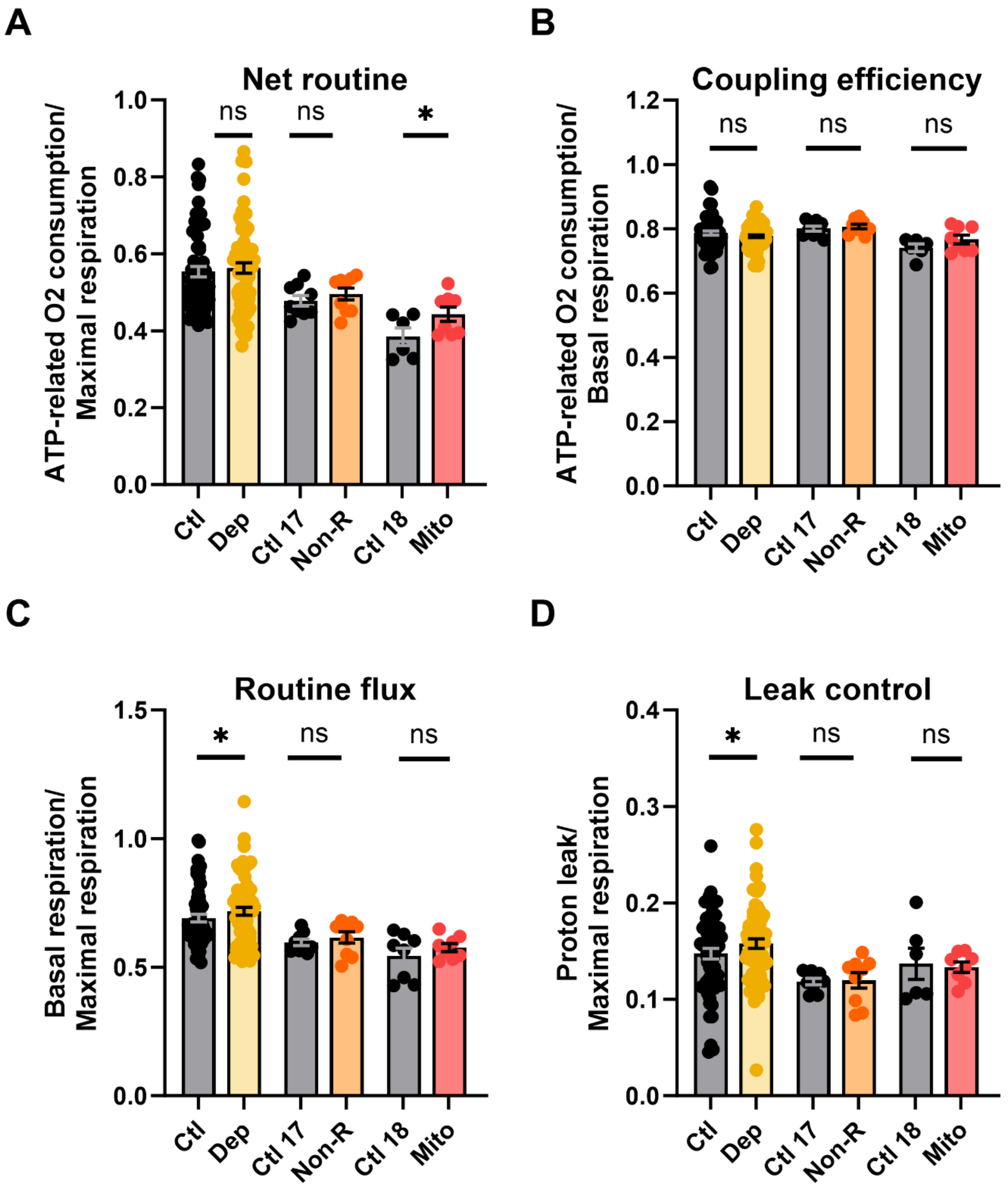

Figure S5. Mitochondrial flux ratios in NPCs. (A) Net routine is the ratio between ATP-related O_2 consumption and maximal respiration **(B) Coupling efficiency** is the ration between ATP-related O_2 consumption and basal respiration **(C) Routine flux** is the ratio between basal and maximal respiration **(D) Leak control** is the ratio between proton leak and maximal respiration. Dot plots show the respective ratios ± SEM Ctl: non-depressed controls cohort; MDD: major depressive disorder cohort; Ctl 17 and Ctl 18: non-depressed controls; Non-R: non-responder patient; Mito: mitochondriopathy patient. All data were analyzed with paired t-test, significant differences were indicated with * ($p<0.05$), ** ($p<0.005$), *** ($p<0.0005$)

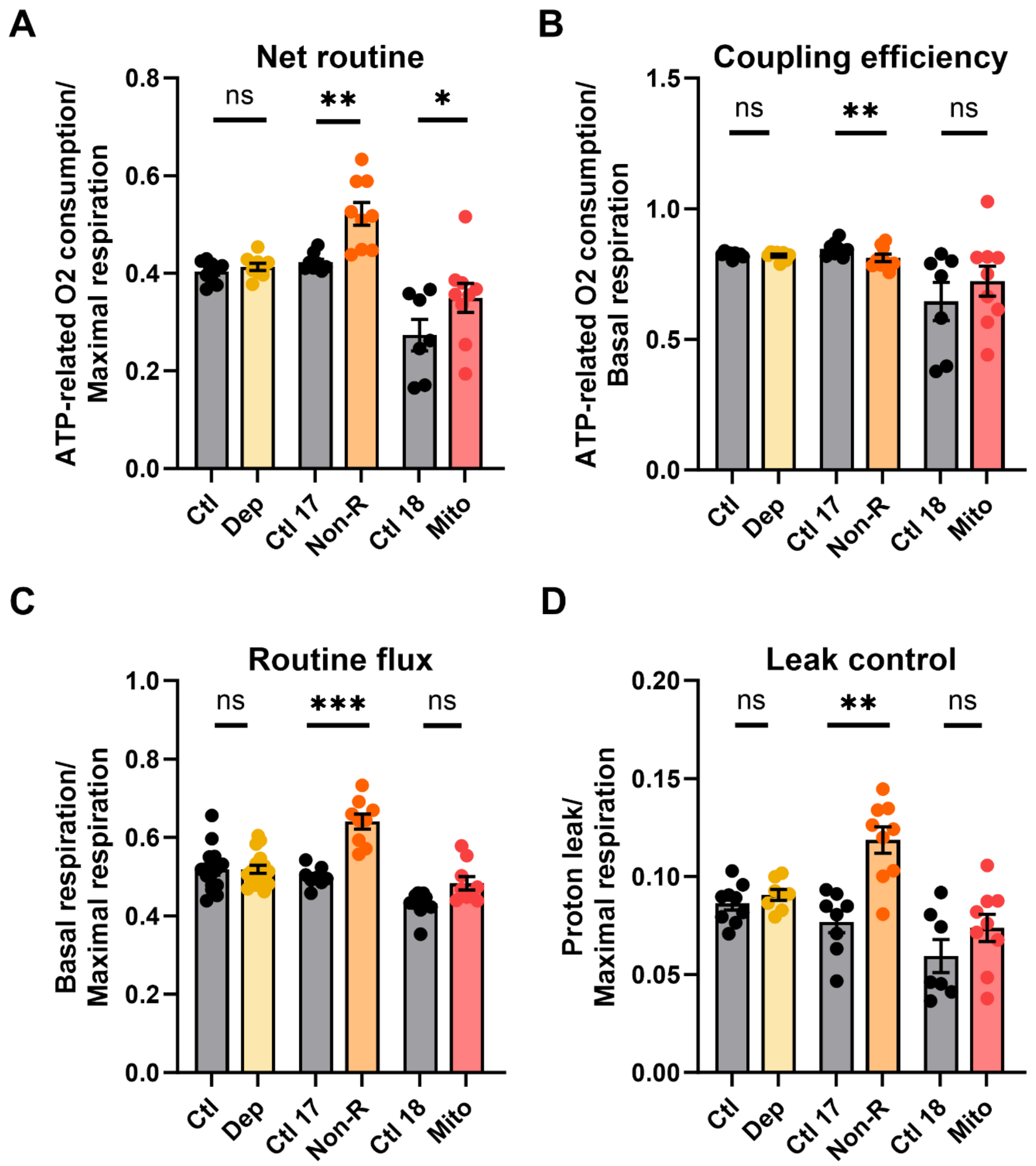

Figure S6. Mitochondrial flux ratios in astrocytes. (A) Net routine is the ratio between ATP-related O_2 consumption and maximal respiration **(B) Coupling efficiency** is the ration between ATP-related O_2 consumption and basal respiration **(C) Routine flux** is the ratio between basal and maximal respiration **(D) Leak control** is the ratio between proton leak and maximal respiration. Dot plots show the respective ratios ± SEM Ctl: non-depressed controls cohort; MDD: major depressive disorder cohort; Ctl 17 and Ctl 18: non-depressed controls; Non-R: non-responder patient; Mito: mitochondriopathy patient. All data were analyzed with paired t-test, significant differences were indicated with * ($p<0.05$), ** ($p<0.005$), *** ($p<0.0005$)

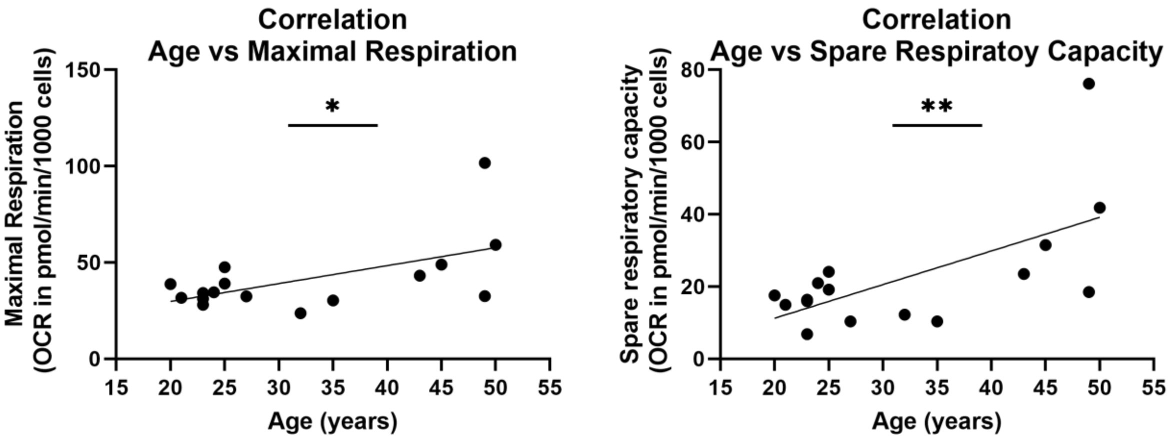

Figure S7. Correlation between age and mitochondrial respiration. Graphs represent Pearson correlation between respiratory data in the non-depressed control cohort (n = 16) and a simple linear regression. Maximal respiration vs age p = 0.0223, Spare Respiratory Capacity vs age p = 0.0093.

Each dot on the graphs represents an individual. Significant differences were indicated with * ($p<0.05$).

Table S1. Mitochondrial bioenergetics in fibroblasts

	Ctl	MDD	Ctl 17	Non-R	Ctl 18	Mito
**Basal respiration** (pmol/min/1000 cells)	18.45±0.47 n=140	15.81±0.41 n=140	15.17±0.54 n=9	19.81±1.04 n=9	15.20±0.58 n=9	13.69±0.75 n=9
	p<0.0001		p=0.039		p=0.1393	
**Maximal respiration** (pmol/min/1000 cells)	36.28±1.11 n=140	29.57±0.82 n=140	44.46±1.26 n=9	67.34±3.77 n=9	40.20±2.17 n=9	32.36±1.17 n=9
	p<0.0001		p=0.0006		p=0.0053	
**Proton leak** (pmol/min/1000 cells)	2.65±0.10 n=140	2.34±0.09 n=140	2.07±0.39 n=9	3.67±0.38 n=9	2.43±0.14 n=9	2.07±0.15 n=9
	p=0.0014		p=0.0004		p=0.1125	
**ATP-related O2 consumption** (pmol/min/1000 cells)	15.77±0.41 n=140	13.25±0.31 n=140	13.10±0.31 n=9	16.14±0.98 n=9	12.98±0.60 n=9	11.62±0.63 n=9
	p<0.0001		p=0.0178		p=0.0486	
**Spare respiratory capacity** (pmol/min/1000 cells)	19.09±0.94 n=140	13.93±0.65 n=140	29.29±1.05 n=9	47.52±2.96 n=9	25.00±1.91 n=9	18.67±1.55 n=9
	p<0.0001		p=0.0004		p=0.0035	
**Non-mito. O2 consumption** (pmol/min/1000 cells)	9.54±0.38 n=140	7.93±0.28 n=140	7.06±0.21 n=9	8.61±0.99 n=9	7.46±0.24 n=9	6.57±0.26 n=9
	p<0.0001		p=0.1418		p=0.0315	
**ATP content** (nM/µg/mL)			29.98±2.78 n=8	26.44±4.34 n=8	21.82±1.03 n=6	17.95±1.81 n=6
			p=0.5998		p=0.0895	
**NAD/NADH** (ratio)			7.89±0.80 n=9	7.15±0.95 n=8	7.37±0.79 n=8	6.80±0.65 n=9
			p=0.7491		p=0.4185	
**Mitochondrial content** (mean fluo.)			5942±348 n=4	8572±1212 n=4	5990±965 n=4	6808±1312 n=4
			p=0.0792		p=0.1948	
**MMP** (relative units)	1.55 ±0.01 n=3971	1.37±0.01 n=3718	1.45±0.07 n=92	1.39±0.10 n=93	2.25±0.07 n=148	2.01±0.05 n=142
	p<0.0001		p=0.2982		p<0.0001	
**Cytosolic Ca2+** (relative units)	0.59±0.01 n=1020	0.59±0.01 n=1114	0.62±0.01 n=86	0.63±0.01 n=93	0.62±0.01 n=108	0.60±0.01 n=87
	p=0.1897		p=0.1037		p=0.3228	

**Mito. Ca2+** (relative units)			257.4±5.1 n=93	337.2±5.8 n=100	253.6±5.2 n=99	241.1±3.8 n=93
			p<0.0001		p=0.3904	
**Cell size** (pixels)	2318±27 n=1063	1975±22 n=1105	2806±101 n=80	2124±102 n=87	2660±94 n=94	2301±82 n=83
	p<0.0001		p<0.0001		p=0.0238	
**Cytosolic ROS** (mean fluo.)			21111±4644 n=3	17900±4366 n=3	13811±2288 n=3	16434±4417 n=3
			p=0.5304		p=0.1791	
**Mitochondrial ROS** (mean fluo.)			2063±401 n=5	3321±215 n=5	2107±252 n=5	1993±247 n=5
			p=0.0074		p=0.7043	
**Lipid peroxidation** (pg/mL 8-isoprostane/1000 cells)			0.30±0.04 n=9	0.23±0.03 n=9	0.18±0.01 n=9	0.27±0.03 n=9
			p=0.1037		p=0.0033	
**Glutathione** (GSH/GSSG ratio)			22.9±3.31 n=3	24.5±3.78 n=3	22.1±1.98 n=3	13.1±1.21 n=3
			p=0.7644		p=0.0285	

Table S2. Correlations between depression severity and biological findings

	Non-Mito. O_2 Consumption	Basal Respiration	Maximal Respiration	Proton Leak	ATP - related O_2 Consumption	Spare Respiratory Capacity	MMP	Cytosolic Ca^{2+}	Cell size
Spearman r	-0.422	-0.321	-0.640	-0.309	-0.33	-0.553	0.222	0.416	-0.0771
95% confidence interval	-0,775 to 0,132	-0,724 to 0,245	-0,872 to -0,175	-0,717 to 0,258	-0,728 to 0,235	-0,835 to -0,0394	-0,342 to 0,669	-0,139 to 0,772	-0,567 to 0,453
P (two-tailed)	0.1184	0.2416	0.0118	0.2613	0.2282	0.0349	0.4226	0.1235	0.7848
P value summary	ns	ns	*	ns	ns	*	ns	ns	ns
Significant? (alpha = 0.05)	No	No	Yes	No	No	Yes	No	No	No

Table S3. Neural progenitor cells markers PAX6 and SOX2

	DAPI	SOX2	% SOX2	PAX6	% PAX6
**Ctl 17 #1**	112	97	86.6	106	94.6
**Ctl 17 #2**	88	82	93.2	83	94.3
**Ctl 17 #3**	101	93	92.1	97	96.0
**Ctl 17 #4**	301	272	90.4	286	95.0
**Total**	602	544	90.4	572	95.0
**Non-R #1**	88	78	88.6	81	92.0
**Non-R #2**	101	94	93.1	94	93.1
**Non-R #3**	87	79	90.8	84	96.6
**Total**	878	795	90.5	831	94.6
**Ctl 18 #1**	88	77	87.5	84	95.5
**Ctl 18 #2**	96	88	91.7	91	94.8
**Ctl 18 #3**	101	94	93.1	97	96.0
**Ctl 18 #4**	285	259	90.9	272	95.4
**Total**	570	518	90.9	544	95.4
**Mito #1**	81	74	91.4	76	93.8
**Mito #2**	89	85	95.5	88	98.9
**Mito #3**	80	75	93.8	77	96.3
**Mito #4**	101	94	93.1	96	95.0
**Total**	351	328	93.4	337	96.0

Table S4. Mitochondrial bioenergetics in neural progenitor cells (NPCs)

	Ctl	MDD	Ctl 17	Non-R	Ctl 18	Mito
**Basal respiration** (pmol/min/1000cells)	7.61±0.28 n=63	6.76±0.23 n=70	5.75±0.28 n=9	7.63±0.47 n=9	5.64±0.35 n=8	5.13±0.12 n=8
	p=0.0008		p=0.0143		p=0.1133	
**Maximal respiration** (pmol/min/1000cells)	11.1±0.44 n=63	9.71±0.39 n=70	9.67±0.49 n=9	12.6±0.92 n=9	10.7±0.92 n=8	8.96±0.39 n=8
	p=0.0001		p=0.0197		p=0.0354	
**Proton leak** (pmol/min/1000cells)	1.77±0.08 n=63	1.48±0.05 n=70	1.14±0.06 n=9	1.48±0.13 n=9	1.33±0.06 n=8	1.20±0.08 n=8
	p=0.0007		p=0.0329		p=0.3496	
**ATP-related O2 consumption** (pmol/min/1000cells)	6.67±0.33 n=63	5.31±0.19 n=70	4.61±0.24 n=9	6.14±0.35 n=9	4.31±0.33 n=8	3.93±0.11 n=8
	p<0.0001		p=0.0143		p=0.0903	
**Spare respiratory capacity** (pmol/min/1000cells)	3.73±0.27 n=63	3.00±0.22 n=70	3.92±0.25 n=9	4.93±0.59 n=9	5.02±0.73 n=8	3.84±0.30 n=8
	p=0.0055		p=0.1210		p=0.0403	
**Non-mito. O2 consumption** (pmol/min/1000cells)	3.45±0.13 n=63	3.01±0.11 n=70	2.19±0.13 n=9	2.36±0.16 n=9	2.32±0.12 n=8	2.00±0.15 n=8
	p=0.0007		p=0.2530		p=0.0619	
**ATP content** (nM/µg/mL)			25.7±1.54 n=6	24.1±2.11 n=6	22.6±2.15 n=6	24.9±1.61 n=6
			p=0.5912		p=0.1331	
**NAD/NADH** (ratio)			4.67±0.25 n=3	5.33±0.29 n=3	4.64±0.24 n=3	5.65±0.16 n=3
			p=0.2374		p=0.0288	
**Mitochondrial content** (mean fluo.)			21792±3230 n=9	18292±3811 n=9	20847±3802 n=6	22812±4514 n=6
			p=0.3030		p=0.2756	
**MMP** (relative units)	2.42±0.01 n=7118	2.17±0.01 n=7120	3.22±0.09 n=300	3.22±0.08 n=296	2.38±0.08 n=300	3.33±0.09 n=298
	p=0.148		p=0.9207		p<0.0001	
**Cytosolic Ca2+** (relative units)	0.56±0.01 n=4700	0.57±0.01 n=4706	0.63±0.01 n=492	0.62±0.02 n=457	0.64±0.02 n=501	0.65±0.02 n=513
	p=0.041		p=0.2137		p=0.0001	
**Mito. Ca2+** (relative units)			372.4±4.2 n=420	487.3±5.6 n=452	512.3±6.3 n=551	444.2±4.3 n=534
			p<0.0001		p<0.0001	
**Cell size** (pixels)	190±1.2 n=2866	165±1.1 n=2844	274±4.8 n=491	243±3.4 n=455	261±3.5 n=500	221±3.0 n=523
	p<0.0001		p<0.0001		p<0.0001	

**Cytosolic ROS** (mean fluo.)		1223±184 n=8	1559±283 n=8	1220±192 n=5	1414±140 n=5
		p=0.3286		p=0.1765	
**Mitochondrial ROS** (mean fluo.)		301±34 n=8	267±31 n=8	327±31 n=5	554±51 n=5
		p=0.4287		p=0.0036	
**Lipid peroxidation** (pg/mL 8-isoprostane/1000 cells)		0.00233±0.00012 n=6	0.000942±8.50e-005 n=6	0.00107±0.00011 n=6	0.00180±0.00027 n=6
		p=0.0002		p=0.0186	

Table S5. Patients and controls information – Astrocytes cohort

	Ctl 5	MDD 5	Ctl 10	MDD 10	Ctl 11	MDD 11
**Age (years)**	24	18	20	21	23	23
**Sex**	Male	Male	Male	Male	Male	Male
**Clinical findings**	Absence of past or present depressive or mental disorders<br><br>Absence of documented health conditions	Recurrent depressive disorder, current episode severe with psychotic symptoms (F33.3)	Absence of past or present depressive or mental disorders<br><br>Absence of documented health conditions	Severe depressive episode without psychotic symptoms (F32.2)	Absence of past or present depressive or mental disorders<br><br>Absence of documented health conditions	Severe depressive episode without psychotic symptoms (F32.2)
**Treatment**	None	Bupropion, Mirtazapin	None	Valdoxan	None	Cipralex

Table S6. Mitochondrial bioenergetics in astrocytes

	Ctl	MDD	Ctl 17	Non-R	Ctl 18	Mito
**Basal respiration** (pmol/min/1000cells)	21.8±0.9<br>n=22	19.5±0.8<br>n=24	19.8±1.6<br>n=9	16.7±1.1<br>n=9	19.4±0.5<br>n=9	20.9±1.0<br>n=9
	p=0.0024		p=0.0207		p=0.2087	
**Maximal respiration** (pmol/min/1000cells)	39.6±1.9<br>n=19	37.4±1.8<br>n=22	41.2±4.3<br>n=9	29.9±2.8<br>n=9	45.3±2.4<br>n=9	44.1±1.8<br>n=9
	p=0.0190		p=0.0018		p=0.6975	
**Proton leak** (pmol/min/1000cells)	3.73±0.19<br>n=22	3.07±0.23<br>n=24	3.27±0.29<br>n=9	2.82±0.29<br>n=9	3.42±0.12<br>n=9	3.61±0.16<br>n=9
	p=0.0119		p=0.0903		p=0.2257	
**ATP-related O2 consumption** (pmol/min/1000cells)	18.8±0.8<br>n=24	16.2±0.6<br>n=24	16.6±1.3<br>n=9	13.9±0.9<br>n=9	16.0±0.5<br>n=9	17.3±0.9<br>n=9
	p=0.0033		p=0.0259		p=0.2153	
**Spare respiratory capacity** (pmol/min/1000cells)	18.22±1.2<br>n=20	18.6±1.0<br>n=24	21.4±2.9<br>n=9	13.2±1.9<br>n=9	24.1±1.0<br>n=8	23.2±1.5<br>n=9
	p=0.5654		p=0.0007		p=0.3528	
**Non-mito. O2 consumption** (pmol/min/1000cells)	7.78±0.43<br>n=22	6.28±0.33<br>n=24	7.72±0.62<br>n=9	6.80±0.16<br>n=9	6.17±0.33<br>n=9	5.86±0.49<br>n=9
	p=0.0067		p=0.2284		p=0.4339	
**ATP content** (nM/µg/mL)	36.7±3.03<br>n=14	39.1±2.83<br>n=14	33.9±5.21<br>n=7	29.3±5.80<br>n=4	34.1±5.19<br>n=6	23.9±4.62<br>n=8
	p=0.2442		p=0.1309		p=0.0486	
**Mitochondrial content** (mean fluo.)			20454±3682<br>n=5	15794±1731<br>n=5	31306±5570<br>n=5	20573±4766<br>n=5
			p=0.1248		p=0.1168	
**MMP** (relative units)	4.14±0.07<br>n=888	3.23±0.05<br>n=1196	2.59±0.05<br>n=382	3.69±0.07<br>n=382	1.97±0.07<br>n=240	1.29±0.03<br>n=240
	p<0.0001		p<0.0001		p<0.0001	
**Cytosolic Ca2+** (relative units)	0.54±0.00<br>n=575	0.5324±0.00<br>n=709	0.60±0.00<br>n=343	0.67±0.00<br>n=378	0.57±0.00<br>n=223	0.58±0.00<br>n=235
	p=0.0009		p<0.0001		p=0.0018	
**Mito. Ca2+** (relative units)	201±2<br>n=550	177±1<br>n=693	361±7<br>n=341	316±5<br>n=373	268±3<br>n=228	246±2<br>n=235
	p<0.0001		p<0.0001		p<0.0001	
**Cell size** (pixels)	1110±27<br>n=448	945±21<br>n=469	1288±39<br>n=223	1130±30<br>n=270	1377±48<br>n=159	1538±48<br>n=160
	p<0.0001		p<0.0001		p=0061	

**Cytosolic ROS** (mean fluo.)		4027±518 n=5	2496±100 n=5	2622±75 n=5	3561±340 n=5
		p=0.0359		p=0.0120	
**Mitochondrial ROS** (mean fluo.)		1697±285 n=5	1378±176 n=5	1714±367 n=5	1729±371 n=5
		p=0.3620		p=0.7621	

Table S7. Mitochondrial membrane potential (MMP), calcium homeostasis and dynamics in neurons

	Ctl 17	Non-R	Ctl 18	Mito
**MMP somas** (relative units)	1.20±0.03 n=342	0.895±0.01 n=771	1.08±0.01 n=865	1.15±0.02 n=1075
	p<0.0001		p=0.0002	
**MMP neurites** (relative units)	6.89±0.32 n=166	6.31±0.31 n=156	5.35±0.19 n=155	8.87±0.22 n=201
	p=0.3834		p<0.0001	
**Cytosolic calcium** (relative units)	0.928±0.01 n=372	0.763±0.01 n=302	0.797±0.01 n=837	0.736±0.01 n=661
	p<0.0001		p<0.0001	
**Mitochondrial calcium** (relative units)	344±6 n=453	354±7 n=359	449±6 n=449	316±4 n=493
	p=0.2800		p<0.0001	
**Cell size** (pixels)	131±2 n=374	132±3 n=291	109±1 n=938	107±1 n=670
	p=0.5262		p=0.0117	
**Calcium transients amplitude** (relative units)	0.26±0.00 n=952	0.26±0.01 n=156	0.24±0.00 n=1189	0.23±0.00 n=1255
	p=0.0015		p=0.0843	
**Calcium transients rise time** (ms)	3.02±0.05 n=948	3.25±0.15 n=148	2.39±0.04 n=1056	3.03±0.05 n=1252
	p=0.0048		p<0.0001	
**Calcium transients time constant of decay** (ms)	6.74±0.14 n=736	9.95±0.90 n=115	5.49±0.08 n=961	12.0±0.26 n=994
	p<0.0001		p<0.0001	

Table S8. Electrophysiological properties of patients-derived neurons

	Ctl	MDD	Ctl 17	Non-R	Ctl 18	Mito
**RMP** (mV)	-26.7±1.02 n=161	-23.7±0.84 n=170	-47.9±2.14 n=55	-56.0±1.71 n=48	-53.1±1.36 n=74	-48.4±1.68 n=68
	p=0.0278		p=0.0024		p=0.0428	
**Capacitance** (pF)	18.1±0.9 n=161	15.8±0.8 n=169	12.8±0.4 n=56	9.2±0.4 n=49	11.4±0.4 n=75	9.7±0.3 n=67
	p=0.0330		p<0.0001		p=0.0065	
**INa 0mV current density** (pA/pF)	-33.0±2.5 n=157	-42.6±3.2 n=163	-17.6±3.1 n=47	-9.7±2.5 n=43	-11.7±1.6 n=60	-31.2±3.1 n=58
	p=0.0116		p=0.4348		p<0.0001	
**IK +20mV current density** (pA/pF)	41.4±1.9 n=226	38.2±1.6 n=245	102±5.4 n=50	123±7.3 n=43	91.2±5.7 n=65	118±5.9 n=61
	p=0.2956		p=0.0254		p=0.0015	
**Spontaneous PSCs** (act./not act.)			26/32=81.25%	6/35=17.14%	23/41=56.10%	33/46=71.74%
			p<0.0001		p=0.1786	
**# of PSCs** (mean number)			106±17.7 n=26	8.5±2.48 n=6	23.0±6.2 n=23	30.5±5.7 n=33
			p<0.0001		p=0.4214	
**Spontaneous AP -50mV** (act./not act.)	18/162=11.11%	35/163=21.74%	19/24=79.14%	16/32=50%	17/35=77.14%	29/38=76.32%
	p=0.016		p=0. 0301		p=0.0170	
**# of AP -50mV** (mean number)			63.2±15.9 n=19	12.9±2.09 n=16	34.4±9.18 n=17	63.2±13.8 n=29
			p=0.0240		p=0.0803	
**Spontaneous AP -80mV** (act./not act.)			21/24=87.50%	20/34=58.82%	19/36=52.78%	27/41=65.85%
			p=0.0216		p=0.2563	
**# of AP -80mV** (mean number)			20.1±5.58 n=21	12.4±2.4 n=20	23.8±6.83 n=19	34.5±8.92 n=27
			p=0.4184		p=0.7273	
**Max amplitude spont. AP** (mV)			-8.09±0.58 n=314	-9.54±0.77 n=127	-10.3±0.48 n=283	-4.11±0.38 n=779
			p<0.0001		p<0.0001	

**Spont. AP FWHM** (ms)		5.94±0.14 n=247	5.22±0.14 n=124	6.09±0.13 n=234	5.25±0.07 n=738
		p<0.0001		p<0.0001	
**PSC amplitude** (pA)		-59.4±0.54 n=2045	-121.2±16.5 n=54	-55.9±0.9 n=634	-39.5±0.6 n=353
		p=0.006		p<0.0001	
**PSC rise time** (ms)		0.432±0.00 n=1994	0.469±0.01 n=50	0.462±0.01 n=691	0.765±0.02 n=372
		p=0.025		p<0.0001	
**PSC time constant of decay** (ms)		1.23±0.01 n=1866	1.07±0.07 n=49	1.22±0.02 n=630	2.27±0.07 n=337
		p=0.7987		p<0.0001	
